# Reduced G protein signaling despite impaired internalization and **β**-arrestin recruitment in patients carrying a CXCR4^Leu317fsX3^ mutation causing WHIM syndrome

**DOI:** 10.1172/jci.insight.145688

**Published:** 2023-03-08

**Authors:** Rajesh Kumar, Samantha Milanesi, Martyna Szpakowska, Laura Dotta, Dario Di Silvestre, Anna Maria Trotta, Anna Maria Bello, Mauro Giacomelli, Manuela Benedito, Joana Azevedo, Alexandra Pereira, Emilia Cortesao, Alessandro Vacchini, Alessandra Castagna, Marinella Pinelli, Daniele Moratto, Raffaella Bonecchi, Massimo Locati, Stefania Scala, Andy Chevigné, Elena M. Borroni, Raffaele Badolato

**Affiliations:** 1 “Angelo Nocivelli” Institute for Molecular Medicine, University of Brescia, Brescia, Italy.; 2 Rheumatology and Clinical Immunology, Azienda Socio Sanitaria Territoriale (ASST) Spedali Civili, Brescia, Italy.; 3 Department of Medical Biotechnology and Translational Medicine, University of Milan, Milan, Italy.; 4 IRCCS Humanitas Research Hospital, Rozzano, Milan, Italy.; 5 Department of Infection and Immunity, Immuno-Pharmacology and Interactomics, Luxembourg Institute of Health, Esch-sur-Alzette, Luxembourg.; 6 Department of Pediatrics, ASST Spedali Civili, Brescia, Italy.; 7 Department of Clinical and Experimental Sciences, University of Brescia, ASST Spedali Civili, Brescia, Italy.; 8 Institute for Biomedical Technologies–National Research Council (ITB-CNR), Segrate, Milan, Italy.; 9 Microenvironment Molecular Targets, Istituto Nazionale Tumori–IRCCS–Fondazione G. Pascale, Naples, Italy.; 10 Department of Clinical Hematology, Pediatric Hospital, Centro Hospitalar e Universitário de Coimbra, Coimbra, Portugal.; 11 Department of Biomedical Sciences, Humanitas University, Pieve Emanuele, Milan, Italy.

**Keywords:** Cell Biology, Immunology, Chemokines, Signal transduction

## Abstract

WHIM syndrome is an inherited immune disorder caused by an autosomal dominant heterozygous mutation in CXCR4. The disease is characterized by neutropenia/leukopenia (secondary to retention of mature neutrophils in bone marrow), recurrent bacterial infections, treatment-refractory warts, and hypogammaglobulinemia. All mutations reported in WHIM patients lead to the truncations in the C-terminal domain of CXCR4, R334X being the most frequent. This defect prevents receptor internalization and enhances both calcium mobilization and ERK phosphorylation, resulting in increased chemotaxis in response to the unique ligand CXCL12. Here, we describe 3 patients presenting neutropenia and myelokathexis, but normal lymphocyte count and immunoglobulin levels, carrying what we believe to be a novel Leu317fsX3 mutation in CXCR4, leading to a complete truncation of its intracellular tail. The analysis of the L317fsX3 mutation in cells derived from patients and in vitro cellular models reveals unique signaling features in comparison with R334X mutation. The L317fsX3 mutation impairs CXCR4 downregulation and β-arrestin recruitment in response to CXCL12 and reduces other signaling events — including ERK1/2 phosphorylation, calcium mobilization, and chemotaxis — all processes that are typically enhanced in cells carrying the R334X mutation. Our findings suggest that, overall, the L317fsX3 mutation may be causative of a form of WHIM syndrome not associated with an augmented CXCR4 response to CXCL12.

## Introduction

WHIM syndrome (OMIM 193670) is a rare inherited disorder of immunity caused by autosomal dominant heterozygous mutations in *CXCR4*. The WHIM acronym refers to the main features of the clinical phenotype, including warts, hypogammaglobulinemia, recurrent bacterial infections, and neutropenia/leukopenia with myelokathexis (peripheral neutropenia due to impaired egress of neutrophils from the bone marrow) (1–3). However, the complete phenotype of the syndrome has been observed in only about 20% of patients. While neutropenia and lymphopenia with myelokathexis are the most consistent features of the disease, hypogammaglobulinemia and warts have been observed in 58% and 61%, respectively, of the patients in our previously reported cohort (4). In addition, both the severity and frequency of infections are highly variable, ranging from patients with almost asymptomatic disease, to patients who develop recurrent pulmonary infections leading to bronchiectasis, or treatment-refractory warts progressing to HPV-related malignancies.

Although WHIM syndrome was first described in 1964 (5), the association of CXCR4 mutations with this syndrome was only found in 2003 (6). All mutations to date identified in WHIM patients affect amino acids of the carboxy-terminal domain of CXCR4, resulting in enhanced and prolonged responses to its unique ligand CXCL12 (7–15). The most common mutation of the disease, R334X, which has been identified in approximately 50% of subjects, and other nonsense or indel mutations (i.e., S338X, p.S339fs342X, p.S341fs365X, p.G323fs343X) all result in the partial truncation of the intracellular tail of CXCR4 (16). In a single family, a missense non-truncating mutation (p.E343K) has been reported to be associated with a milder phenotype, impaired CXCR4 downregulation, and reduced CXCR4 desensitization (17).

The cytoplasmic tail of CXCR4 can deliver signals through both G protein–dependent and –independent pathways (12). CXCR4 has 18 potential serine/threonine phosphorylation sites in its 50–amino acid C-terminal domain (18). These sites have diverse roles in the normal functioning of CXCR4, including the uncoupling of the ligand-activated receptor from G proteins and activation of the G protein–independent signaling pathways (7–9, 19–22). Moreover, GPCR kinase–mediated phosphorylation of CXCR4 leads to the recruitment of β-arrestins, consequently inducing clathrin-dependent internalization, ubiquitination, and degradation of the receptor. It has been demonstrated in patients with WHIM syndrome that GRK6 and β-arrestin-2 recruitment is impaired (8, 10, 11), leading to the gain-of-function CXCR4 activity responsible for the abnormal cellular trafficking of the receptor.

We describe what we believe to be a novel CXCR4 mutation, Leu317fsX3, associated with a form of WHIM syndrome, characterized by neutropenia and myelokathexis, but normal lymphocyte count and immunoglobulin levels. Analysis of CXCR4^Leu317fsX3^ function revealed unexpected signaling behavior, as the mutation prevents both β-arrestin recruitment and receptor internalization upon CXCL12 stimulation but also reduces the extent of G protein signaling, calcium mobilization, ERK phosphorylation, and chemotaxis. These findings highlight the diversity of the causative mutations in WHIM syndrome, their impact on the receptor functions, and the complex pathogenesis of this rare genetic disorder.

## Results

### Genetic examination of CXCR4^Leu317fsX3^ mutation causing WHIM syndrome.

**Figure 1:**
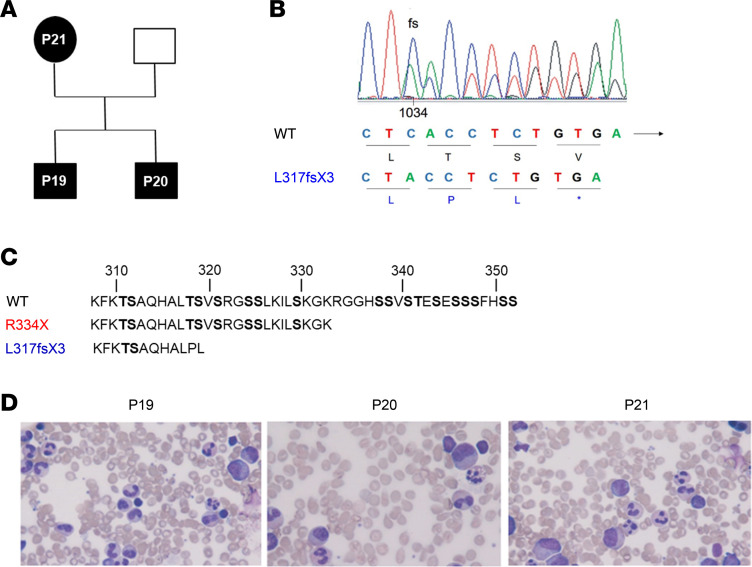
Genetic and bone marrow analysis of WHIM patients with CXCR4^L317fsX3^ mutation. (**A**) Pedigree of WHIM patients, consistent with autosomal dominant inheritance. (**B**) Nucleic acid alignment showing the deletion of cytosine 1034 and histogram showing the frameshift (fs) in the CXCR4 ORF. (**C**) Amino acid sequence of the CXCR4 C-terminal domains in CXCR4^WT^ and CXCR4^R334X^ and CXCR4^L317fsX3^ mutants. (**D**) Original magnification of images is ×40. Bone marrow aspirate from WHIM patients showing full myeloid maturation in the phase of peripheral neutropenia, consistent with myelokathexis.

We identified 3 Portuguese patients (named P19, P20, and P21 in our WHIM database) with clinical history and laboratory findings suggesting the diagnosis of WHIM syndrome (Figure 1A and Table 1). Sanger sequencing of the *CXCR4* gene in these subjects identified a cytosine deletion at nucleotide 1034 (c.1034delC) causing a shift in the ORF with a change in the amino acid sequence creating a new stop codon after 2 amino acids (L317fsX3) (Figure 1B), thereby truncating 33 of the 50 residues of the receptor C-terminal tail (Figure 1C). This also removes all but two (T311 and S312) of the 18 serine/threonine residues, which are important phosphorylation sites for the correct functioning of the receptor.

### Clinical manifestations and leukocyte distribution in CXCR4^L317fsX3^ WHIM syndrome.

**Figure 2:**
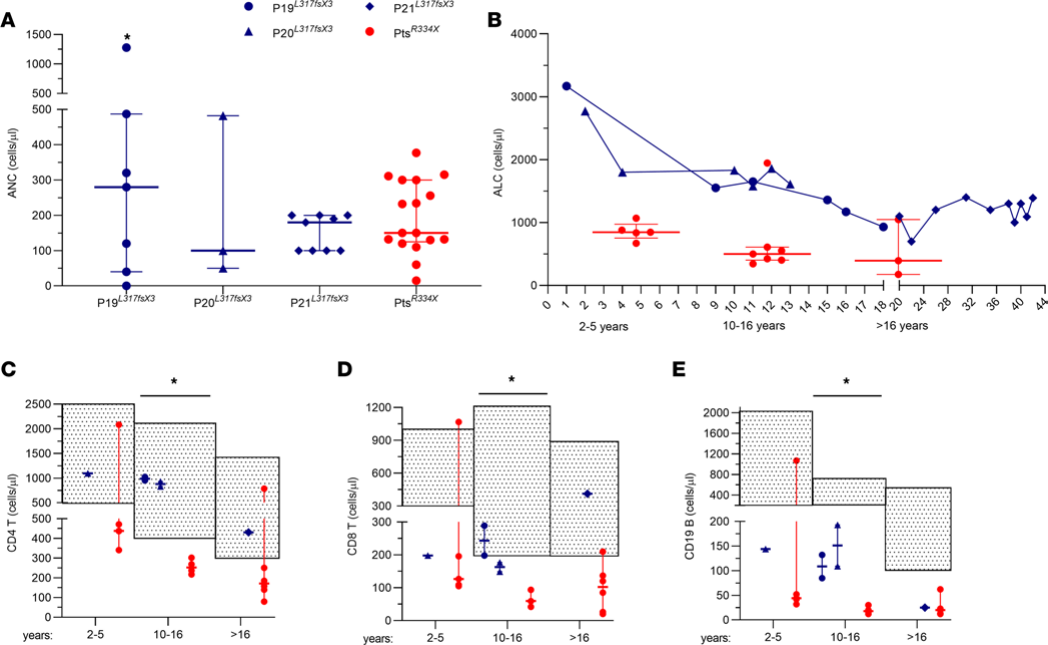
Comparative analysis of blood counts in CXCR4^L317fsX3^ and CXCR4^R334X^ WHIM patients. (**A**) Absolute neutrophil counts (ANCs) of CXCR4^L317fsX3^ WHIM patients during clinical follow-up as compared with ANCs of CXCR4^R334X^ WHIM patients at clinical onset. Values are shown as median with interquartile range. *ANC value in P1 during rhinovirus infection. (**B**) Absolute lymphocyte counts (ALCs) of CXCR4^L317fsX3^ WHIM patients since diagnosis compared with age-matched CXCR4^R334X^ WHIM patients (for ages 2–5 years, 10–16 years, and >16 years/adulthood). Values are shown as median with interquartile range. (**C**–**E**) Absolute counts of CD4^+^ (**C**), CD8^+^ (**D**), and CD19^+^ (**E**) lymphocyte subsets in CXCR4^L317fsX3^ WHIM patients (blue; we analyzed 2 tests for both P19 and P20 for the 10–16 age group, 1 test for P20 for the 2–5 age group, and 1 test for P21 for the >16 age group) compared with age-matched CXCR4^R334X^ WHIM patients (red; for the CD4^+^ T lymphocytes we analyzed 5 R334X patients for the 2–5 age group, 4 for the 10–16 age group, and 6 for the >16 age group; for the CD8^+^ T lymphocytes we considered 3 R334X patients for the 2–5 age group, 1 for the 10–16 age group, and 2 for the >16 age group; for CD19^+^ B cells we considered 5 R334X patients for the 2–5 and >16 age groups and 4 R334X patients for the 10–16 age group). The fill pattern represents the age-matched normal range. The analysis of L317fsX3 patients compared with the R334X patients for the age group 10–16 years resulted in a significant difference between the 2 groups in the severity of lymphopenia for the CD4^+^, CD8^+^, and CD19^+^ subsets (*P < 0.014).

The patient designated as P19 was an 11-year-old boy referred for evaluation following an episode of hallux cellulitis treated with oral antibiotics. The review of past medical records revealed that he was first found to be neutropenic at the age of 2 years, with an absolute neutrophil count (ANC) below 500 cells/μL, and only upper respiratory tract infections. On presentation, his physical examination was unremarkable. Laboratory tests revealed severe neutropenia (ANC 40 cells/μL), associated with leukopenia (WBCs 1,490 cells/μL) and monocytopenia (absolute monocyte count 90 cells/μL); however, the lymphocyte count was normal for his age (absolute lymphocyte count 1,360 cells/μL, age-matched range 1,000–5,300 cells/μL). Bone marrow aspirates showed pathological features with hyperplasia of the myeloid cellular line, hypersegmented granulocytes with cytoplasmic vacuoles and low granules, all suggestive of myelokathexis (Figure 1D). During his follow-up when free of infection, ANC ranged from 40 to 282 cells/μL (Figure 2A); absolute monocyte count was from 60 to 153 cells/μL, while absolute lymphocyte count ranged from 930 to 1,589 cells/μL (Figure 2B). During rhinovirus infection, cell counts increased to normal values (WBCs 2,460 cells/μL, ANC 1,280 cells/μL, and absolute monocyte count 240 cells/μL). Analysis of lymphocyte subsets showed inconsistent lowering of CD4^+^ or CD8^+^ T lymphocytes but a persistently low B cells count (total B lymphocytes ranged from 57 to 132 cells/μL; age-matched range 200–600 cells/μL) (Figure 2, C–E). Serum immunoglobulin levels were in the normal range (Table 1). At 14 years of age, a skin wart of his hand was observed, which responded to initial topical treatment. There was evidence of relapse, but this resolved with cryotherapy.

The 4-year-old brother of the proband (designated P20) presented with a single episode of pneumonia when he was 1 year of age. On that occasion, neutropenia was first detected (ANC 400 cells/μL, WBCs 2,000 cells/μL). Past medical history revealed he had recurrent otitis media. Laboratory tests confirmed severe neutropenia (ANC 100 cells/μL), but normal total lymphocyte count (absolute lymphocyte count 1,800 cells/μL, age-matched range 1,700–6,400 cells/μL) and normal CD3^+^ and CD4^+^ T cells despite reduction of CD8^+^ T cells (148 cells/μL, age-matched range 300–1,600 cells/μL) (Figure 2D) and B cells (total B cells 87 cells/μL, age-matched range 200–2,100 cells/μL) (Figure 2E). During his follow-up, ANC ranged from approximately 44 to 102 cells/μL (Figure 2A), absolute monocyte count from 88 to 123 cells/μL, and absolute lymphocyte count from 1,127–1,830 cells/μL (normal count for >5 years is >1,100 cells/μL) (Figure 2B). At time of writing, he had not developed hypogammaglobulinemia. Bone marrow aspirate was consistent with myelokathexis (Figure 1D). He had a single skin hand wart at the age of 9 years successfully treated with cryotherapy without recurrence. The 2 patients did not display signs of pulmonary disease and did not require treatment with granulocyte colony-stimulating factor, nor intravenous immunoglobulin. The infectious episodes were successfully treated with standard course of antibiotics.

When their mother (designated P21) was investigated, a past medical history revealed leukopenia with neutropenia since the age of 15 years (detected following phlebitis from minor wound). As for her infectious history, she had been suffering from chronic sinusitis since the age of 10 years presented with periodontal disease at the age 16 years. She only had a single wart on her finger at 15 years that resolved spontaneously, and since then a regular HPV screening program has been normal. Her bone marrow aspirate at the age of 25 years revealed pathological features compatible with myelokathexis (Figure 1D). During her follow-up, ANC remained below 200 cells/μL, with normal total lymphocyte count between 1,090 and 1,400 cells/μL; however, at time of writing she had never developed hypogammaglobulinemia, nor pulmonary disease or recurrence of warts. Interestingly, total lymphocyte count tends to decrease with age in WHIM patients with R334X mutation as well as in those with the CXCR4^Leu317fsX3^ mutation. This is possibly related to reduced thymic output or migration to lymphoid organs with age. However, total lymphocyte counts were consistently higher in the L317fsX3 WHIM patients (Figure 2B). The CD4^+^, CD8^+^, and CD19^+^ lymphocyte counts of the 3 patients carrying the Leu317fsX3 mutation were significantly higher than levels measured in WHIM patients with R334X mutation (Figure 2, C–E).

### CXCR4^L317fsX3^ shows impaired CXCL12-induced receptor downregulation.

**Figure 3:**
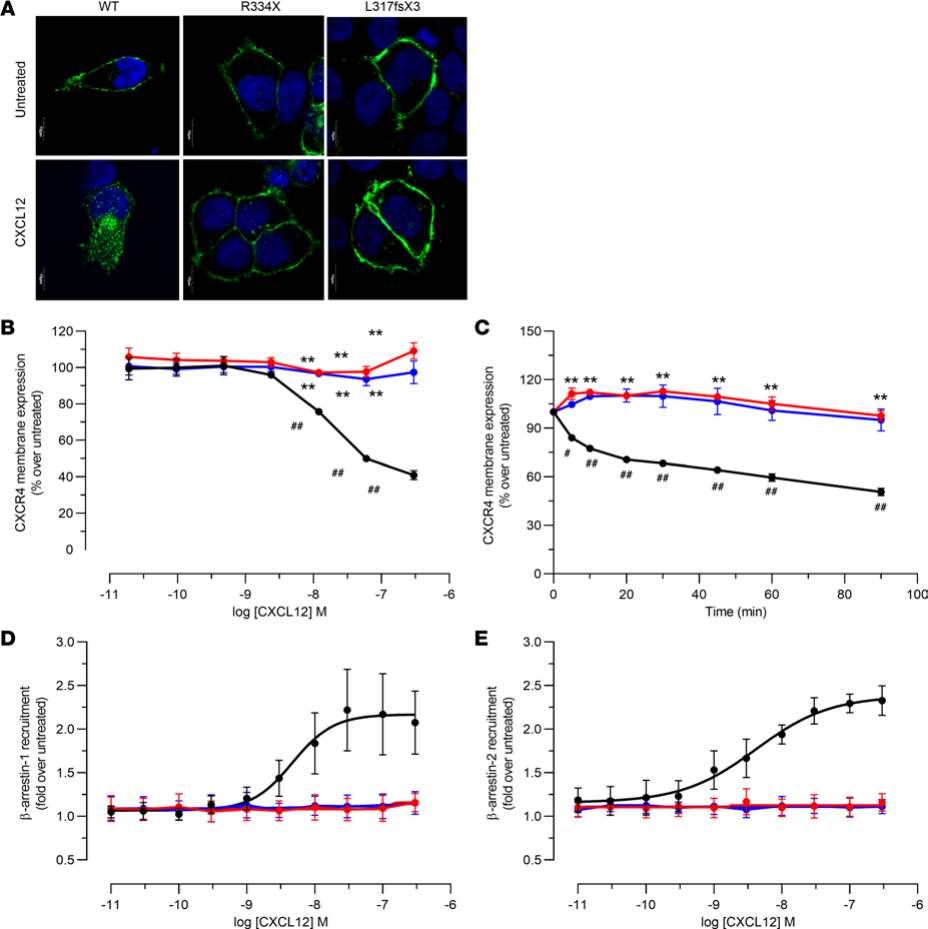
Differential internalization and β-arrestin recruitment profiles of CXCR4^WT^ and WHIM-mutated variants. (**A**) HEK293T cells were transiently transfected with GFP-tagged WT and WHIM-mutated CXCR4. (**A**) Confocal images of cells treated with vehicle or 100 nM CXCL12 for 60 minutes. For each panel, blue represents nuclear staining (DAPI), whereas green stains for CXCR4. Scale bar: 10µm. (**B** and **C**) HEK293T cells were transiently transfected with CXCR4^WT^ and mutated variants N-terminally tagged with HiBiT. Panels show bioluminescence quantification of CXCR4 membrane expression upon treatment with 0.02–300 nM CXCL12 for 90 minutes (**B**) or 50 nM CXCL12 for the indicated times (**C**). (**D** and **E**) HEK293T cells were transiently transfected with CXCR4^WT^ and mutated variants C-terminally tagged with SmBiT and β-arrestins N-terminally tagged with LgBiT. Recruitment of β-arrestin-1 (**D**) and β-arrestin-2 (**E**) to CXCR4 receptors was evaluated upon treatment with 0.01–300 nM CXCL12 for 20 minutes. Data are expressed as percentage over untreated cells and are shown as mean ± SEM of 3 independent experiments. **P < 0.0001, WHIM-mutated variants versus WT; ^#^*P* < 0.05, ^##^*P* < 0.0005, CXCL12 vs. untreated. Black lines, CXCR4^WT^; red lines, CXCR4^R334X^; blue lines, CXCR4^L317fsX3^.

GPCR stimulation with an agonist usually leads to a reduction of receptor membrane level following internalization, which is an important step in the process known as downregulation. As previous reports showed that CXCR4 downregulation was impaired by WHIM mutations (23), we tested the effect of the L317fsX3 in these processes and compared it with WT receptor or receptor bearing the most common WHIM mutation, R334X. First, basal and CXCL12-dependent distribution of WT and WHIM-mutated receptors was visualized by confocal microscopy (Figure 3A). In basal conditions, all receptor variants were predominantly present at the plasma membrane (Figure 3A, top panels). Upon stimulation with CXCL12, cell surface CXCR4 was significantly reduced for the WT receptor, with distinguishable vesicle-like structures accumulating within intracellular compartments. In contrast, both CXCR4^R334X^ and CXCR4^L317fsX3^ persisted at the cell surface following chemokine stimulation, suggesting impaired receptor internalization (Figure 3A, bottom panels). Receptor internalization was thus further analyzed with a highly sensitive nanoluciferase complementation–based cell surface detection approach using CXCR4^WT^ and WHIM-mutated receptors N-terminally tagged with HiBiT (24, 25). We monitored CXCR4 surface expression by measuring light emitted by the different HiBiT-tagged receptors in the presence of recombinant LgBiT and NanoLuc substrate. Notably, all receptors exhibited similar levels of surface expression (Supplemental Figure 1A; supplemental material available online with this article; https://doi.org/10.1172/jci.insight.145688DS1), which allowed us to quantitatively compare their internalization following ligand stimulation. We could show that the membrane level of CXCR4^WT^ was markedly reduced both in concentration-response (Figure 3B) and kinetics (Figure 3C) experiments. Conversely, the surface expression levels of CXCR4^R334X^ and CXCR4^L317fsX3^ remained unchanged after CXCL12 stimulation (Figure 3, B and C), suggesting an impaired downregulation.

Given that downregulation of GPCR requires a coordinated response involving β-arrestins, the ability of the WHIM mutant to recruit β-arrestins following CXCL12 stimulation was examined (26). Interactions of β-arrestins with WHIM-mutated CXCR4 have been previously investigated, with discrepant findings (8, 10). CXCR4^WT^ and WHIM-mutated receptors C-terminally tagged with SmBiT and β-arrestins N-terminally tagged with LgBiT were used to investigate the impact of WHIM mutations on these interactions. All transfectants exhibited similar levels of CXCR4 surface expression, as revealed by flow cytometry using mAb 12G5 (Supplemental Figure 1B), which allowed us to quantitatively compare the recruitment of β-arrestins to these receptors. Results showed that in response to increasing concentrations of CXCL12, CXCR4^WT^ efficiently recruited β-arrestin-1 and β-arrestin-2, while the 2 WHIM mutations prevented the recruitment of both β-arrestins to CXCR4 (Figure 3, D and E). These data were consistent with the clustering of all known mutations causing WHIM syndrome in the C-terminal domain of CXCR4, which contains serine and threonine residues involved in the phosphorylation of cytoplasmic elements critical for β-arrestin recruitment (18).

### CXCR4^L317fsX3^ shows preserved Gα_i_-mediated signaling but reduced CXCL12 responsiveness.

Impaired downregulation of C-terminus–mutated CXCR4 after agonist stimulation is commonly postulated to account for the increased receptor signaling and function, which was proposed as a pathogenic mechanism operating in WHIM syndrome (18). Therefore, we investigated the effect of L317fsX3 mutation on CXCR4 interactions with G proteins and activation of downstream signaling.

**Figure 4:**
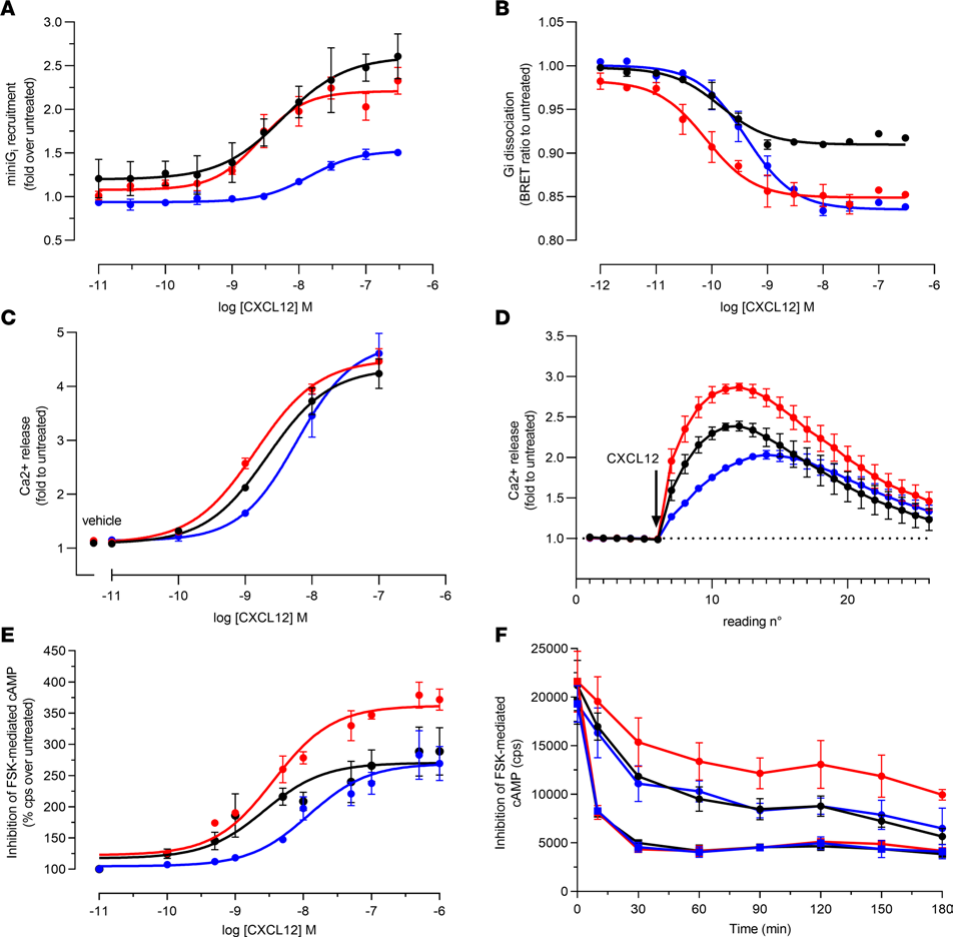
Comparison of the G protein interaction, activation, and signaling of CXCR4^WT^ and WHIM-mutated variants. (**A**) HEK293T cells were transiently transfected with SmBiT-tagged CXCR4^WT^ and WHIM-mutated variants, and LgBiT-tagged miniG_i_. Recruitment of miniG_i_ to CXCR4 was evaluated upon treatment with 0.01–300 nM CXCL12 for 20 minutes. (**B**) HEK293T cells were transiently transfected with nanoluciferase-tagged Gα subunit and Venus-tagged Gβγ dimer. G protein subunit dissociation following WT or WHIM CXCR4 variant activation was evaluated as BRET signal upon treatment with CXCL12 at indicated concentrations. (**C** and **D**) HEK293T cells were transfected with CXCR4-encoding pIRES vector, calmodulin C-terminally fused to SmBiT, and MYLK2S N-terminally fused to LgBiT vectors. Calcium flux was evaluated over 2 minutes by luminescence upon stimulation with CXCL12. Results are presented as concentration-response curves (**C**) and signal evolution over time for CXCL12 at 1 nM (**D**). (**E** and **F**) HEK293T cells were stably transfected with HA-tagged CXCR4^WT^ and WHIM-mutated variants. cAMP levels were evaluated by luminescence for AlphaScreen signal upon treatment with 0.1–1000 nM CXCL12 for 30 minutes (**E**) or 200 nM CXCL12 (circles) and vehicle (squares) for the indicated times (**F**). cps, count per second. Data are expressed as fold (**A**, **C**, and **D**) or percentage (**E**) over untreated cells and are shown as mean ± SEM of 2 (**E**) and 3 (**A**–**D** and **F**) independent experiments. EC_50_ and E_max_ were obtained by nonlinear regression curve fitting using GraphPad software analysis (Table 2). Black lines, CXCR4^WT^; red lines, CXCR4^R334X^; blue lines, CXCR4^L317fsX3^.

CXCR4^WT^ and WHIM-mutated receptors C-terminally tagged with SmBiT (Supplemental Figure 1B) were first used to assess their ability to interact with the G protein α subunit by monitoring of CXCL12-induced recruitment of LgBiT-tagged miniG_i_ protein (27). We observed that compared with CXCR4^WT^, CXCR4^R334X^ displayed a similar profile in response to CXCL12 with slightly improved potency (Figure 4A and Table 2). In contrast, both the efficacy and potency were reduced toward CXCR4^L317fsX3^, suggesting that its shorter C-terminal tail impairs the optimal interaction with the α subunit of G protein.

To compare the ability of the different CXCR4 variants to activate G proteins, we next applied a NanoBRET assay monitoring dissociation of heterotrimeric G proteins from untagged receptors upon chemokine stimulation. Results indicated that all 3 receptors could couple and activate G proteins. Notably, the amplitude of the signal of G protein dissociation was higher for CXCR4^R334X^ and CXCR4^L317fsX3^ compared with CXCR4^WT^, in line with the observed lack of receptor downregulation (Figure 3). In agreement with the miniG_i_ recruitment data, CXCL12 displayed a 2-fold stronger potency toward CXCR4^R334X^ compared with CXCR4^WT^, consistent with the reported gain-of-function profile of WHIM mutations (10), whereas a 3-fold weaker potency was observed toward CXCR4^L317fsX3^ (Figure 4B and Table 2).

To further evaluate the impact of L317fsX3 mutation on G protein signaling, we monitored the modulation of downstream secondary messengers. Chemokine-induced increase of intracellular calcium ions was assessed using NanoBiT-based assays and untagged receptors. In line with the G protein dissociation data, CXCL12 displayed a 2-fold stronger potency toward CXCR4^R334X^ compared with CXCR4^WT^, while an opposite effect on G protein activation was again observed for CXCR4^L317fsX3^ (Figure 4C and Table 2). The differences between the variants were further evidenced by the kinetics analysis, which revealed a marked increase in calcium release for CXCR4^R334X^, while the signal was both reduced and delayed for CXCR4^L317fsX3^ in comparison with CXCR4^WT^ (Figure 4D and Table 2).

The Gα_i_-driven inhibition of forskolin-induced accumulation of intracellular cAMP after stimulation with CXCL12 was also analyzed using CXCR4^WT^ and WHIM-mutated stable transfectants that exhibited similar levels of surface CXCR4 expression when analyzed by anti-HA staining (Supplemental Figure 1C). CXCL12 displayed similar efficacy in reducing cAMP via CXCR4^WT^ and CXCR4^L317fsX3^, whereas a significantly higher response was observed for CXCR4^R334X^. We again observed a reduction in CXCL12 potency for CXCR4^L317fsX3^ compared with CXCR4^WT^ and CXCR4^R334X^, reminiscent of G protein and calcium data (Figure 4E and Table 2).

Finally, to evaluate the effect of WHIM mutations on the duration of CXCR4 activation, the wane in cAMP-dependent signal was also monitored over time. While CXCR4^L317fsX3^ followed a trend in intracellular cAMP levels similar to that of CXCR4^WT^, CXCR4^R334X^ showed a higher Gα_i_-dependent signaling that lasted over time (Figure 4, E and F, and Supplemental Figure 2).

### CXCR4^L317fsX3^ is not a gain-of-function mutation.

**Figure 5:**
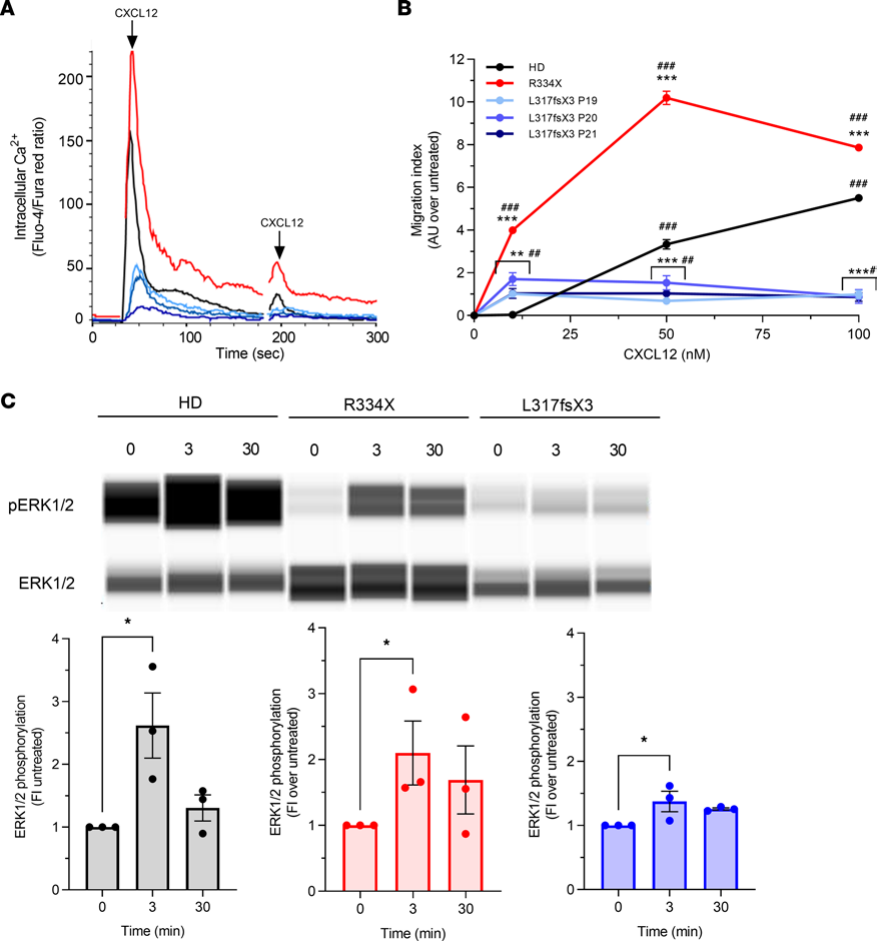
Functional and signaling properties of CXCR4^WT^ and WHIM-mutated variants. (**A** and **B**) PHA-activated T cells derived from fresh PBMCs of healthy donor (HD) and thawed PBMCs of WHIM patients (P). (**A**) Flow cytometry quantification of intracellular calcium upon treatment with 5 nM CXCL12 added at 30 and 180 seconds (indicated with arrows). Data are from 1 experiment representative of *n* = 2 performed and are expressed as ratio of Fluo-4 over Fura Red. (**B**) Cell migration upon treatment with CXCL12. Data are expressed as migration index over untreated and are shown as mean ± SEM of 3 independent experiments. **P < 0.005, ***P < 0.0001, WHIM-mutated variants versus WT; ^#^*P* < 0.05, ^##^*P* < 0.005, ^###^*P* < 0.0001, CXCL12 vs. untreated. (**C**) Western blot analysis and quantification of ERK1/2 phosphorylation upon treatment with 100 nM CXCL12 for indicated times in EBV-transformed cells derived from 1 PBMC of healthy donor and WHIM patients, 1 from each genotype. Data are shown as mean ± SEM of 3 independent experiments. *P < 0.05. Black bars/lines, healthy donor; red bars/lines, WHIM-mutated CXCR4^R334X^ WHIM patient; blue bars/lines, CXCR4^L317fsX3^ WHIM patient.

We next investigated the functional effects of L317fsX3 mutation in patient-derived phytohaemagglutinin (PHA)-activated T cells, testing calcium flux and chemotaxis in response to CXCL12. In all three CXCR4^L317fsX3^ patients, flow cytometry quantification of Fluo-4/Fura Red ratio showed reduced calcium flux in comparison with the healthy donor and CXCR4^R334X^, the latter displaying an enhanced response (Figure 5A), supporting previous reports and the results described above (Figure 4D) (28). A similar pattern was observed following a second reactivation of CXCR4. Consistent with this, migration of PHA-T cells was also reduced at high CXCL12 doses in all three CXCR4^L317fsX3^ patients compared with healthy donor and CXCR4^R334X^, the latter again displaying a greater response when compared with CXCR4^WT^ (Figure 5B). Because of technical limitations, we were unable to measure CXCR4 expression levels in PHA-T cells, which could also influence the extent of their response to CXCL12. However, similar results were obtained in K562 cells stably transfected with both CXCR4 WT and WHIM variants expressed at equal level, suggesting that the effect observed in chemotaxis with patient-derived cells stems from the intrinsic signaling properties of the receptors (Supplemental Figure 7).

To evaluate the impact of WHIM mutations on CXCR4 signaling pathways that underpin the receptor’s functional properties, we took advantage of phosphoprotein array to analyze the phosphorylation profile of a broad spectrum of kinases and their downstream signaling proteins in WT and WHIM-mutated EBV cells at short (3 minutes) and prolonged (30 minutes) stimulation times with CXCL12 (Supplemental Figure 3). As compared with the effects of CXCR4^WT^, WHIM mutations profoundly affected the phosphorylation profile induced by CXCL12. The activation of several downstream kinases observed with CXCR4^WT^ was impaired (i.e., P53, RSK, PYK2, STAT1, WNK) or abrogated (i.e., SRC, WNK, β-catenin, STAT6) in both CXCR4^R334X^ and CXCR4^L317fsX3^ cells. Based on these findings, our signaling network model depicted a scenario of general downregulation of phosphorylation for both CXCR4 WHIM-mutated variants (Supplemental Figures 4–6 and Supplemental Tables 2 and 3). Nevertheless, several kinases were specifically upregulated downstream of CXCR4^R334X^ (i.e., AKT1/2/3 [S473] and HSP60) and CXCR4^L317fsX3^ (i.e., CHK-2 and HSP27). However, a significant number of kinases showing substantial alterations in phosphorylation extent and kinetics in CXCR4^R334X^ were not affected in CXCR4^L317fsX3^ and appeared comparable to CXCR4^WT^. Interestingly, some kinases with peak values reached at a short time point for the WT receptor were switched off (i.e., P53, GSK-3α/β, RSK1/2/3) or kept active until a longer time point in CXCR4^R334X^ (i.e., ERK1/2). In line with these findings, Western blot analysis confirmed prolonged ERK1/2 phosphorylation in CXCR4^R334X^ as compared with healthy donor and one CXCR4^L317fsX3^ patient (Figure 5C). Comparable results for L317fsX3 mutation were obtained in HEK293T cells that exhibited similar levels of surface CXCR4 expression upon HA staining (Supplemental Figure 8). The reconstructed networks further analyzed by Gene Ontology showed that the most enriched pathways are involved in “signal transduction,” “endocrine system,” and “immune system” (Supplemental Figure 9A), and a topological analysis that underpinned EGFR, ERK1/2, P53, and PDGFRβ found them to be the best-ranked proteins by betweenness (Supplemental Figure 9B and Supplemental Table 3), suggesting a role as hubs. Moreover, upon CXCR4 mutations, IL-8, LCK, JUN, and EGFR were predicted as the most affected nodes, as they showed higher value of interference; specifically, IL-8, LCK, and JUN decreased their betweenness value, while it increased for EGFR (Supplemental Figure 9C and Supplemental Table 2).

## Discussion

In the present study, we describe what we believe to be a novel heterozygous frameshifting mutation, L317fsX3, in the C-terminus of CXCR4 identified in a single Portuguese family with the classical WHIM syndrome autosomal dominant pattern of inheritance.

The clinical manifestations of WHIM syndrome are extremely heterogeneous; moreover, the cases reported so far have not permitted a genotype-phenotype correlation. Even mutations that are closely located to the L317fsX3 mutation, which we describe, may have completely distinct clinical features, including severe phenotype as reported for S319Cfs mutation (29).

As usually observed in most WHIM patients, the manifestation of the disease in the three CXCR4^L317fsX3^ patients was marked by the detection of neutropenia and leukopenia during their hospitalization for infections. Myelokathexis was present in all 3 patients, but the severity of infections was often mild. Notably, the 2 siblings presented with infections requiring hospitalization only when the disease was diagnosed, while the following episodes were mild and were treated with antibiotics at home. In their mother, the most remarkable manifestation was chronic sinusitis, without a history of recurrent pneumonia nor evidence of other complicated infections during her life. In addition, for the characterization of this mutation, we have compared the lymphocyte counts in the three CXCR4^L317fsX3^ patients with those of patients carrying the R334X mutation, which is diagnosed in more than 50% of WHIM patients. Interestingly, we observed that all CXCR4^L317fsX3^ patients displayed normal total lymphocyte counts from early childhood to adult life besides the physiological decline of lymphocyte counts as observed in healthy subjects (30). This observation suggests that lack of lymphopenia might also result in a lower rate of infections, including those sustained by HPV.

In the CXCR4^L317fsX3^ patients, we observed reduction of peripheral B lymphocyte counts, as is usually observed in patients with WHIM syndrome, while the number of recent bone marrow emigrants, as marked by CD38^hi^CD21^dim/lo^CD27^–^ staining, was normal. In addition, P20 showed a defect in CD27^+^ memory or naive B cells, while P21 displayed a normal distribution of B cell subsets, which can reflect the observation that none of the CXCR4^L317fsX3^ patients displayed hypogammaglobulinemia.

Like other known mutations causing WHIM syndrome, L317fsX3 affects the C-terminal domain of CXCR4, where a frameshift removes the last 33 amino acids, including 16 of the 18 serine and threonine residues. These residues generate the so-called GPCR phosphorylation barcode that determines β-arrestin recruitment to CXCR4 and drives its downregulation and desensitization (31). To date, the role of β-arrestins in WHIM remains to be elucidated (32). The signaling profile of WHIM-mutated CXCR4 has currently been explored for 5 mutations (R334X, G336X, S338X, E343K, L329fs341X), whereas signaling properties of remaining variants are presently largely unknown. Studies have been conducted in either ex vivo settings using freshly isolated leukocytes or CD34^+^ cells from patients, and immortalized cells (EBV), or in vitro experimental settings using several cell lines. Our results in HEK293T cells are consistent with the pull-down data published by McCormick et al. in HeLa cells that demonstrated a defective recruitment of β-arrestin-2 to CXCR4^R334X^ after CXCL12 stimulation (10). However, the pull-down data published by Lagane et al. demonstrated a preserved β-arrestin-2 interaction with CXCR4 in leukocytes isolated from WHIM patients carrying the S338X mutation (8). These results underline the complexity of investigating the molecular basis accounting for altered β-arrestin–dependent signaling of CXCR4 in WHIM patients. We have demonstrated that the L317fsX3 mutation impairs ligand-induced receptor internalization, which is consistent with the lack of β-arrestin recruitment. However, the L317fsX3 does not result in a gain-of-function defect. The expected enhancement of CXCL12-induced signaling that has been observed so far in all reported WHIM-mutated CXCR4 and here reported for CXCR4^R334X^ is surprisingly obliterated in CXCR4^L317fsX^; thus it retains milder G protein–dependent signaling. These results are consistent with the role of the full CXCR4 C-terminal domain that, although not essential, is important for efficient Gα_i_ activation (12). Therefore, it is conceivable that, differently from the herein described L317fsX3 mutation, in the R334X variant the additional 14 C-terminal amino acids (319 to 333) themselves or other unknown associated molecules facilitate the binding, conformational change, and activation of interacting Gα_i_ upon CXCL12 stimulation that lead to the gain-of-function activity in the absence of receptor downregulation. Furthermore, several observations in the literature could suggest the involvement of other mechanisms, such as the heterodimerization of WHIM-mutated CXCR4 with WT receptors (8, 33) and the effect on survival/apoptosis (34, 35), that deserve further investigations. The reason why the phenotype of WHIM may largely vary remains to be explored. Our observations suggest that in the case of the variant L317fsX3, normal total lymphocyte count together with the lower rate of infectious manifestations, warts in particular, is explained by the lack of an exaggerated CXCR4 signaling.

The GPCR signaling appears to be more complex than expected (36). GPCRs were proposed to generate G protein–independent functions, including the functional network involving β-arrestins (37). Our results show how WHIM mutations profoundly affected the phosphorylation profile of several activated kinases downstream of CXCR4: both R334X and L317fsX3 variants may impair or abrogate the phosphorylation status. However, the pattern of phosphorylation impairment differs for these WHIM variants, as for some kinases the L317fsX3 remains comparable to the WT, at variance with R334X. Interestingly, CXCR4^R334X^, but not CXCR4^L317fsX3^, also showed kinetic differences, with short time peak values of some kinases that were switched off or maintained at extended times. Interestingly, ERK1/2 signaling emerged as a key hub in CXCR4 signaling downstream of WHIM-mutated variants, consistently with the reported ERK1/2 signaling dysfunction that has already been observed in CXCL12-activated leukocytes or cell lines carrying CXCR4 WHIM-mutated variants R334X and S338X in both WHIM and Waldenström macroglobulinemia patients (32).

The CXCR4 antagonist plerixafor has been investigated in a phase I trial in 3 WHIM patients carrying CXCR4^R334X^ for 6 months (38), and in an open-label study with 3 severely affected patients for 19 to 52 months (39). This targeted treatment successfully ameliorated myelofibrosis, panleukopenia, and infections, particularly HPV manifestations. More recently, a phase II trial with the oral selective CXCR4 antagonist mavorixafor has proved beneficial in terms of cellular mobilization (40). Conversely, our results show how WHIM syndrome can be caused by CXCR4 variants that do not behave as gain-of-function mutations, underlying the need for functional characterization of novel genetic variants to explore patient-targeted therapeutic strategies.

In conclusion, the present study provides new insights into the molecular and biochemical basis of complex WHIM syndrome phenotypes. Though also affecting the C-terminal domain of the receptor, the newly identified L317fsX3 mutation does not lead to an increased ligand-induced receptor responsiveness and signaling in contrast to the previously identified WHIM-causing mutations. These data clearly demonstrated that the interpretation of WHIM syndrome as the consequence of excessive CXCR4-dependent G protein–dependent signaling activity sustained by gain-of-function mutations for an impaired negative regulation of arrestins appears limited. Furthermore, most of our information refers to signaling properties of most common CXCR4 mutations and their impact on the receptor chemotactic activity, while properties of emerging variants and their effects on CXCR4 activities are presently largely unknown and deserve further investigations. Long-term follow-up will be necessary to evaluate whether the clinical manifestations observed in patients carrying the L317fsX3 mutation confer a favorable clinical evolution of this phenotype.

## Methods

### Patients.

We obtained written informed consent from all patients or, when a minor, their parent, and the study was conducted under the ASST Spedali Civili of Brescia Ethical Committee protocol (WHIM-02 NP 2874). Data from the 3 Portuguese patients reported were collected through a disease-based questionnaire as part of the WHIM syndrome database, whose results have already been partly published (4). None of the other siblings presented neutropenia. The immunological parameters, analyzed by both the Pediatric Hospital of Coimbra and the Institute for Molecular Medicine “Angelo Nocivelli”, were compared with the reference values of the herein-mentioned Institute for Molecular Medicine “Angelo Nocivelli” that conducted the study, based on a database obtained from a pool of age-matched healthy subjects.

### Genetic testing.

DNA was isolated from peripheral blood using QIAamp DNA Mini Kit (Qiagen) and quantified with an Infinite M200 UV/visible spectrophotometer (Tecan). Sanger sequencing of CXCR4 was carried out in the 3 patients with clinical features of WHIM syndrome on an ABI PRISM 310 Genetic Analyzer (Applied Biosystems), and the files were analyzed with BioEdit software (41). Primers are listed in the Supplemental Materials.

### Cell lines.

HEK293T (ATCC) and K562 transfectants (obtained from the National Cancer Institute’s Developmental Therapeutics Program) were grown in DMEM and RPMI, respectively, with 10% FBS, 1% HEPES, and 1% penicillin-streptomycin (Lonza) and 800 μg/mL geneticin (Invitrogen) were added to stable transfectants. Transfectant cell lines were generated using polyethylenimine (PEI) at a ratio of 3:1 (PEI:DNA) or Lipofectamine 2000 (Invitrogen) for HEK293T cells, and TransFectin Lipid Reagent (Bio-Rad Laboratories) for K562 cells, according to the manufacturers’ instructions. PBMCs were isolated from peripheral blood using Lympholyte Cell Separation Media (Euroclone).

EBV immortalized cells were obtained from 1 × 10^6^ PBMCs incubated for 18 hours at 37°C with supernatant derived from monkey cotton-top tamarin B lymphoblastoid B95-8 cell line (Sigma-Aldrich) and resuspended in 1 mL RPMI complete medium with 15 μg/mL PHA. For PHA-activated T cells, 3 × 10^5^ PBMCs/mL were seeded in RPMI 1640 with 10% FBS, 2 mM L-glutamine, 1 U/mL penicillin, 1 μg/mL streptomycin, 600 U/mL IL-2, and 5 μg/mL PHA for 48 hours.

### Confocal microscopy analysis.

GFP-tagged HEK293 transient transfectants (1 × 10^5^) were seeded onto glass discs, stimulated with 100 nM CXCL12 (R&D Systems) for 60 minutes in DMEM with 1% BSA (Sigma-Aldrich), and fixed with 4% paraformaldehyde for 15 minutes. Coverslips were incubated with DAPI (Molecular Probes) for 5 minutes, and mounted with FluoSave (Calbiochem). High-resolution images (512 × 512 pixels) were acquired sequentially with a ×60 1.4 NA Plan-Apochromat oil immersion objective using an FV1000 laser scanning confocal microscope (Olympus).

### Flow cytometry analysis.

One hundred to two hundred microliters whole blood was stained for immunophenotypic analysis using standard multiparametric flow cytometry protocols consisting of a combination of monoclonal antibodies (Becton Dickinson) according to the manufacturer’s instructions, and further analyzed by FlowJo software version 8.8.7 (Tree Star). Transiently transfected HEK293T cells were stained for CXCR4 expression for 1 hour with anti-CXCR4 antibody (clone 12G5, 1:20) and the isotype control, both from BD Pharmingen, and both directly conjugated with PE (Supplemental Figure 1B). HEK293T cells transfected with HA- or pEGFP-tagged receptors were stained for 1 hour with 5 μg/mL anti-HA primary antibody (Covance) and 1 μg/mL Alexa Fluor 647–conjugated goat anti-mouse IgG (Molecular Probes) secondary antibody (Supplemental Figure 1C and Supplemental Figure 8A). K562 cells were stained with PE-conjugated anti-CXCR4 monoclonal antibody 12G5 (R&D Systems). PE-conjugated mouse IgG2a κ isotype (BD Biosciences) was used as negative control (Supplemental Figure 7A). Data were acquired by BD FACSCanto Flow Cytometer and LSRFortessa Flow Cytometer and analyzed by BD FACSDiva and FlowJo software version 10.7.1 (Becton Dickinson).

### Receptor internalization analysis.

CXCR4 surface levels were monitored by NanoLuc complementation assay using the Nano-Glo HiBiT extracellular detection system (Promega), according to the manufacturer’s protocol, in 1.2 × 10^6^ HEK293T cells transiently transfected with 100 ng plasmid encoding CXCR4^WT^ or WHIM mutants N-terminally tagged with HiBiT, a small part of the nanoluciferase with high affinity toward LgBiT. CXCR4 internalization induced by 50 nM CXCL12 for the indicated times or CXCL12 at concentrations ranging from 20 pM to 300 nM for 90 minutes was evaluated per light emission from complementation of LgBiT protein with remaining surface receptor–fused HiBiT and determined using a Mithras LB940 plate reader (Berthold Technologies) (24).

### β-Arrestin and miniG_i_ protein recruitment to CXCR4.

CXCL12-induced β-arrestin or miniG_i_ protein recruitment to CXCR4 was monitored by NanoLuc complementation assay (NanoBiT, Promega) as previously described (42–44). 5 × 10^6^ HEK293T cells were cotransfected with pNBe vectors encoding WT or WHIM mutant CXCR4 C-terminally tagged with SmBiT and human β-arrestins (arrestin-2 and -3) or miniG_i_ protein (engineered GTPase domains of Gα_i_ subunits) N-terminally tagged with LgBiT (45). Twenty-four hours after transfection, cells were harvested and incubated for 15 minutes at 37°C with Coelenterazine-H (Regis Technologies). One hundred thousand cells per well were then distributed into white 96-well plates. β-Arrestin and miniG_i_ recruitment to the receptor induced by CXCL12 at concentrations ranging from 10 pM to 300 nM was evaluated for 20 minutes with a Mithras LB940 luminometer (Berthold Technologies). EC_50_ and E_max_ were obtained by nonlinear regression curve fitting using GraphPad software analysis.

### G protein dissociation assay.

The measurement of Gα_i_ protein dissociation upon receptor activation was performed using NanoBRET-based assay (46). HEK293T cells were plated in a 6-well plate (0.8 × 10^6^ per well) and cultured for 24 hours before transfection with a polycistronic vector encoding the Gα subunit of G proteins fused to the nanoluciferase and the Gβγ dimer fused to a circular permutated Venus fluorescent protein (Addgene 168120). Twenty-four hours after transfection, cells were harvested, incubated for 3 minutes at 37°C with Coelenterazine-H (Regis Technologies), and distributed into white 96-well plates, each well containing 1.5 × 10^5^ cells. The BRET signal generated was measured with a GloMax plate reader (Promega) using a 450 BP filter for the donor luminescence and a 530 LP filter for the fluorescent acceptor signal.

### cAMP modulation assay.

cAMP levels were measured by AlphaScreen cAMP assay (Perkin Elmer) according to the manufacturer’s instructions in HEK293T CXCR4^WT^ and WHIM-mutated stable transfectants, generated with lipofection (Lipofectamine 2000, Invitrogen) using HA-tagged pcDNA3 plasmids. Cells were stimulated with indicated concentrations of CXCL12 for 30 minutes. The luminescence of the beads was read on a Synergy H4 Hybrid Microplate Reader (BioTek). Briefly, forskolin (10 μM; Sigma-Aldrich) was added to the cells simultaneously with CXCL12 (200 nM), starting from the longest time point (180 minutes) to the shortest ones up to time zero when no forskolin and CXCL12 were added but the plate was immediately read by the plate reader to detect the luminescence of the beads.

### Calcium flux assay.

In PHA-activated T cells, calcium fluxes were measured by Fluo-4 and Fura Red fluorescent dyes. PHA-activated T cells (1 × 10^6^) were stimulated with 50 nM CXCL12 for 30 and 150 seconds after the start of data acquisition by a FACSCalibur flow cytometer (Becton Dickinson). Results were analyzed using FlowJo software.

In HEK293T cells, an assay based on nanoluciferase complementation (NanoBiT) and Ca^2+^-dependent calmodulin–MYLK2S protein association was used to measure calcium fluxes (47). Cells were plated in a 6-well plate (8 × 10^5^ cells per well) and cultured for 24 hours before transfection with CXCR4-encoding pIRES vectors (100 ng), vectors encoding for calmodulin C-terminally fused to SmBiT (50 ng) and MYLK2S N-terminally fused to LgBiT (50 ng). Twenty-four hours after transfection, cells were incubated in PBS supplemented with 1 mM CaCl_2_ and 0.5 mM MgCl_2_·6H_2_O for 10 minutes at 37°C. Coelenterazine-H (Regis Technologies) was then added, and cells were distributed in a 96-well plate (1 × 10^5^ cells per well) and incubated for 20 minutes at 37°C. The baseline signal was acquired for 2 minutes. Calcium flux upon stimulation with CXCL12 (100 nM) or the calcium ionophore A23187 (1 μM) was quantified using the changes in luminescence measured on a GloMax plate reader. EC_50_ and E_max_ were obtained by nonlinear regression curve fitting using GraphPad software analysis.

### Chemotaxis assay.

Chemotaxis was performed using 5 μm Transwell plates or Boyden chamber for PHA-activated T cells or K562 cell lines. PHA-activated T cells (1 × 10^5^) or K562 cells (2 × 10^5^) were plated in the upper chamber, whereas medium with CXCL12 at indicated concentrations was added in the lower chamber. After 2 hours for PHA-activated T cells and 4 hours for K562 cell lines, migrated cells were counted using a FACSCalibur flow cytometer (Becton Dickinson).

### Phosphokinase assay.

The signaling properties of WT and WHIM-mutated CXCR4 were investigated using a Proteome Profiler Human Phospho-Kinase Array Kit (ARY003C, R&D Systems) according to the manufacturer’s instructions in 600 μg protein lysates from 3 × 10^6^ EBV cells stimulated with 100 nM CXCL12 for 3 and 30 minutes. Array membranes were exposed to x-ray film (ChemiDoc Imaging System, Bio-Rad), and pixel densities were collected and normalized on reference spots with Image Lab software (Bio-Rad). Heatmap plot was generated using pheatmap library (Pretty Heatmaps CRAN package, Raivo Kolde, 2019, https://CRAN.R-project.org/package=pheatmap).

A *Homo sapiens* protein-protein interaction (PPI) signaling network model was reconstructed by matching of the phosphoproteins characterized in this work and the signaling network retrieved by PesCa Cytoscape’s app (48); specifically, activation, inhibition, and docking relations were considered. The reconstructed network was analyzed at topological level by Centiscape Cytoscape’s app (49); betweenness, bridging, and centroid centralities for directed network were calculated to select hub proteins as previously reported (50). Centralities were calculated processing the reconstructed PPI signaling network, with (+) and without (–) the presence of CXCR4; finally, betweenness values were used to calculate the interference value (CXCR4^+^ betweenness – CXCR4^–^ betweenness) (51). Statistical significance of all topological results was tested by consideration of randomized network models; they were reconstructed and analyzed by an in-house R script based on VertexSort (to build random models), igraph (to compute centralities), and ggplot2 (to plot results) libraries. Results were visualized in the form of violin plots.

Functional Annotation Tool of DAVID database (52) was used to characterize the most enriched Kyoto Encyclopedia of Genes and Genomes (KEGG) pathways. Specifically, background = *Homo sapiens*, count > 10, *P* < 0.001, and FDR < 0.01 were set.

### ERK phosphorylation analysis.

The Wes System (ProteinSimple, Bio-Techne) was used according to the manufacturer’s instructions and with Compass software. Briefly, the following reagents were used: EZ Standard Pack (ProteinSimple PS-ST01EZ-8), Anti-Rabbit Detection Module (ProteinSimple DM-001), and 12- to 230-kDa Wes Separation Module (ProteinSimple W004-1). In total, 0.4 μg/μL of protein sample was loaded. The voltage used was 375  V for a separation time of 25 minutes. The incubation time used for the primary and secondary antibodies was 30 minutes each. The following primary antibodies from Cell Signaling Technologies were used: phospho–p44/42 MAPK (Erk1/2) (Thr202/Tyr204) (source: rabbit; 9101) and p44/42 MAPK (Erk1/2) (source: rabbit; 9102).

### Statistics.

Statistical analyses were performed using GraphPad Prism software. The ordinary 1- or 2-way ANOVA test with Dunnett’s multiple comparison, and Kruskal–Wallis with uncorrected Dunn’s test were used for statistical comparisons.

### Study approval.

We obtained written informed consent from all patients or, if a minor, from their parents, and the study was conducted under and with the approval of the ASST Spedali Civili of Brescia Ethical Committee protocol (WHIM-02 NP 2874).

## Author contributions

AMT, AMB, RK, SM, AV, MS, MP, MG, EMB, and A Chevigné performed experiments. MB, JA, AP, EC, LD, and R Badolato followed up with the patients and collected clinical data. SS, RK, SM, DDS, AV, A Castagna, MS, LD, AC, MP, DM, MG, EMB, and A Chevigné analyzed results and made the figures. AC, MS, R Bonecchi, ML, EMB, LD, R Badolato, and A Chevigné designed the research and wrote the paper. RK, SM, and MS are listed as first, second and third, according to the enrollment schedule in the project.

## Supplementary Material

Supplemental data

Supplemental table 1

Supplemental table 2

Supplemental table 3

## Figures and Tables

**Table 1:**
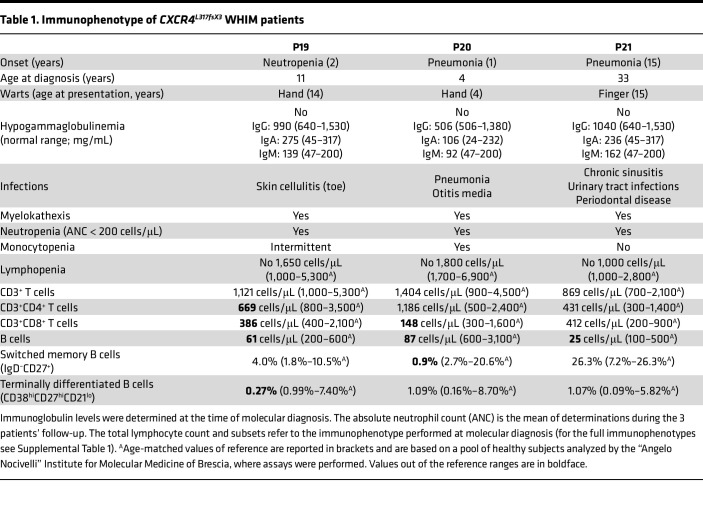
Immunophenotype of *CXCR4^L317fsX3^* WHIM patients

**Table 2:**
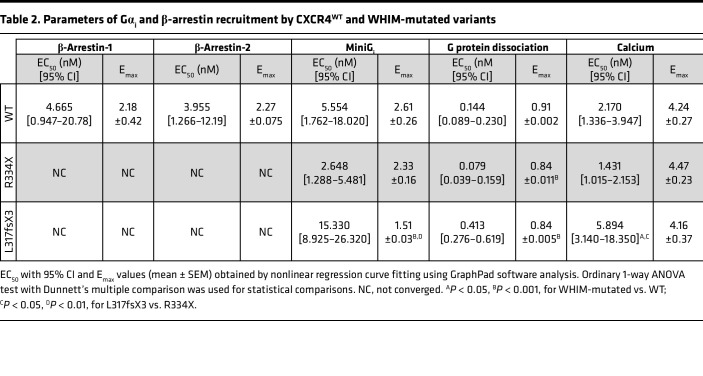
Parameters of Gα_i_ and β-arrestin recruitment by CXCR4^WT^ and WHIM-mutated variants

## Version 1 03/08/2023

Electronic publication
