# Supplementary material for: Reduced G protein signaling despite impaired internalization and β-arrestin recruitment in patients carrying a CXCR4Leu317fsX3 mutation causing WHIM syndrome
Source: JCI Insight. 2023 Mar 8;8(5):e145688. doi: 10.1172/jci.insight.145688 (PMC10077478; doi:10.1172/jci.insight.145688)
Supplement: Supplemental data [file jciinsight-8-145688-s242.pdf]

SUPPLEMENTARY FIGURES AND FIGURE LEGENDS

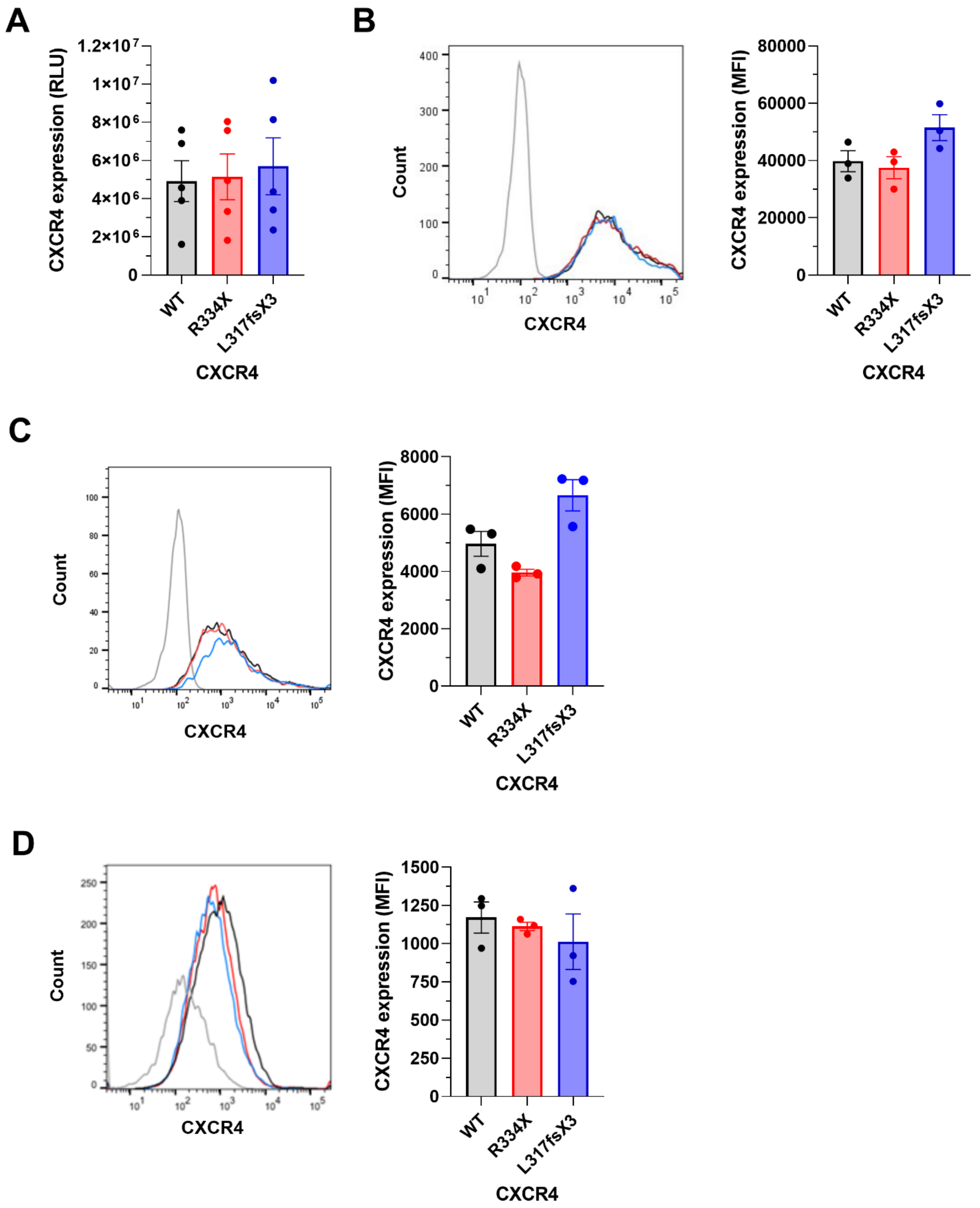

Figure S1. Surface expression of CXCR4^{WT} and WHIM-mutated variants in cell lines.

Surface expression of CXCR4^{WT} and WHIM-mutated receptors in HEK-293T (A-C) transient transfectants used for internalization assay (A; Figure 3B-C), β-arrestin and miniG$_i$ protein

recruitment (B; Figure 3D-E and Figure 4A), and calcium flux assay (C, Figure 4C-D); (D) stable transfectants used for cAMP modulation assay (Figure 4E-F) . Y-axis shows the RLU (A) or mean fluorescence intensity (B-D) of CXCR4 expression reveled by complementation with LgBiT (A) and 12G5 staining (B-D), respectively. $CXCR4^{WT}$: black lines; $CXCR4^{R334X}$: red lines; $CXCR4^{L317fsX3}$: blue lines; isotypic control: gray lines.

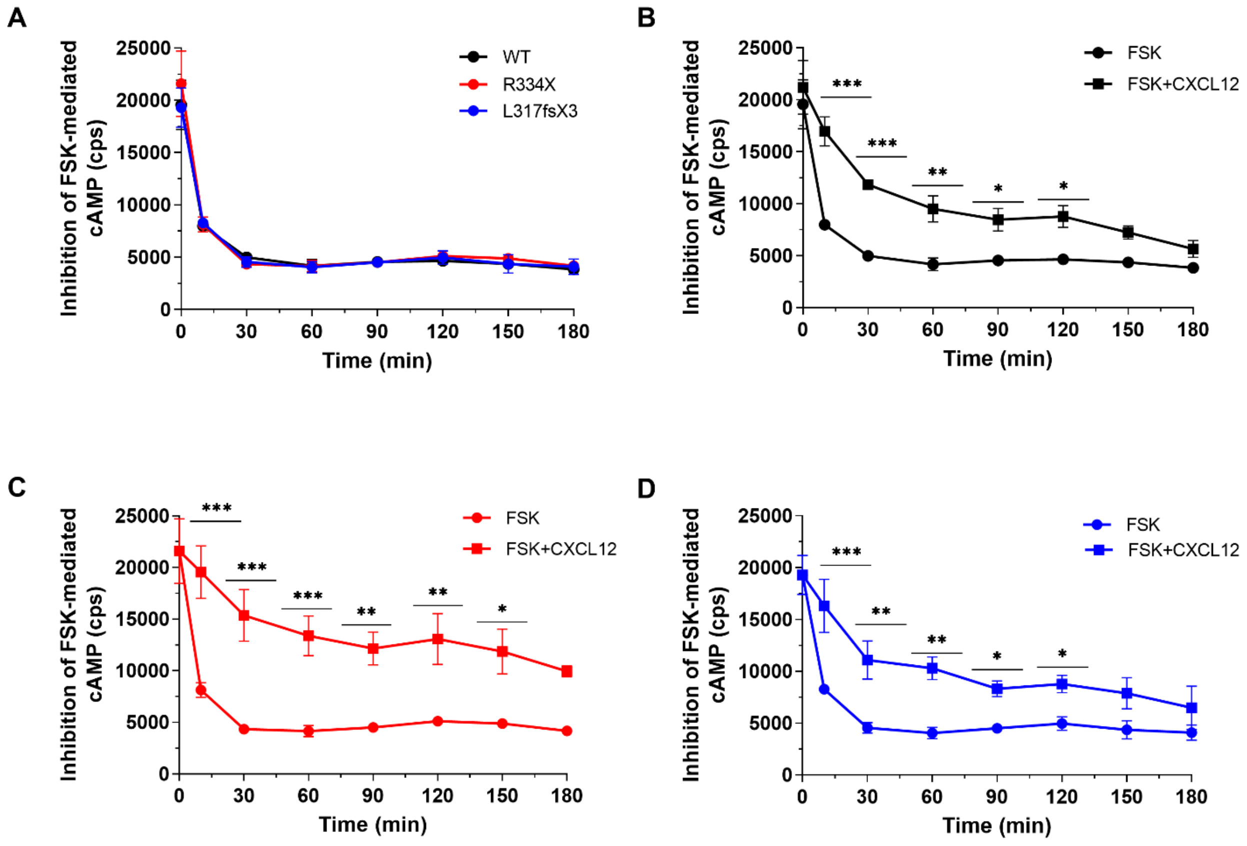

Figure S2. $G_{\alpha i}$ activation properties of CXCR4^{WT} and WHIM-mutated variants.

HEK-293T cells stably transfected with HA-tagged CXCR4^{WT} and WHIM-mutated variants evaluated by luminescence for cAMP levels upon treatment with FSK alone (A, circle dots) or with 200 nM CXCL12 (B-D, square dots) for the indicated time points. Data are shown as mean ± SEM of 3 independent experiments. CXCR4^{WT}: black lines; CXCR4^{R334X}: red lines; CXCR4^{L317fsX3}: blue lines. Two-way ANOVA with Sidak's multiple comparison test was used for statistical analysis. *: $p < 0.05$, **: $p < 0.005$: FSK vs FSK+CXCL12.

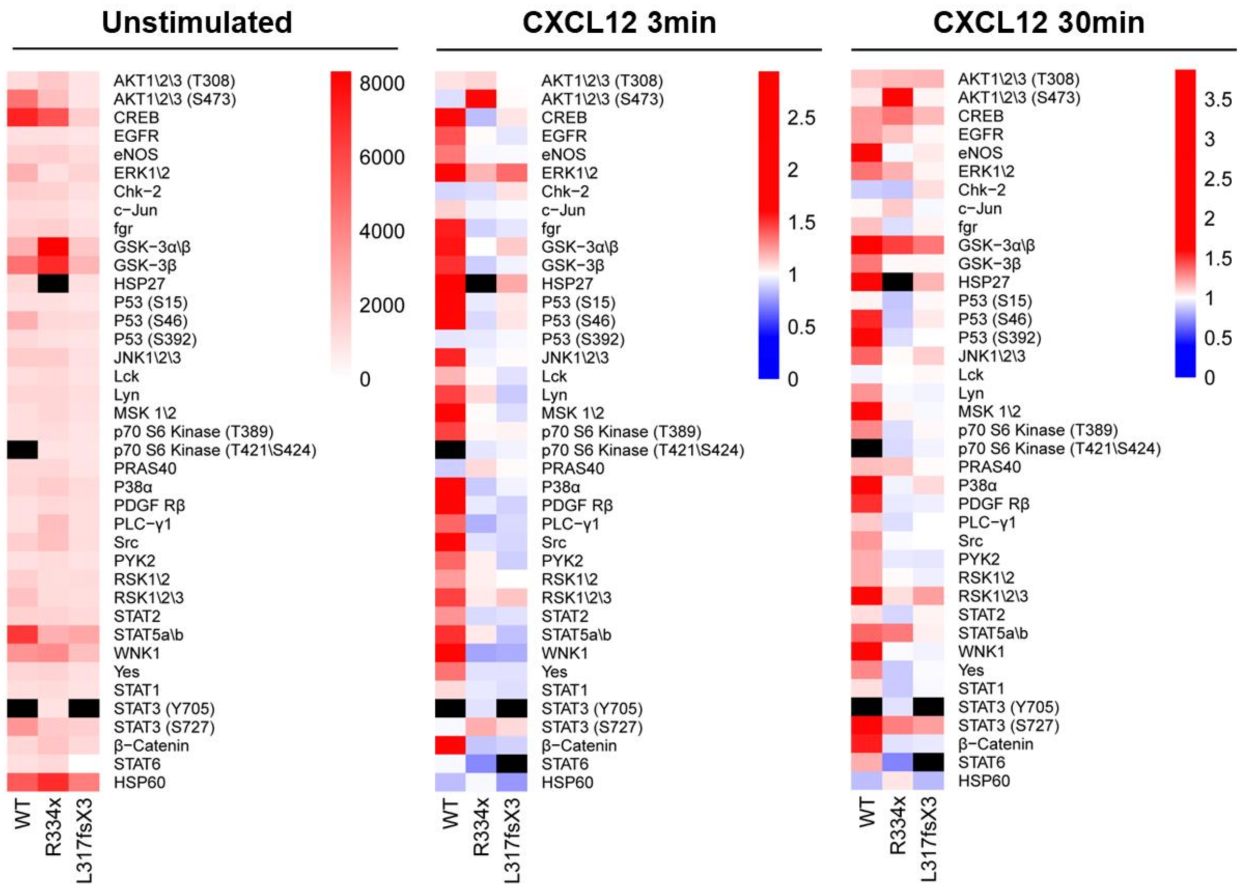

Figure S3. CXCL12-induced signaling pathways in CXCR4^{WT} and WHIM-mutated variants.

EBV transformed cells derived from PBMC of healthy donor and WHIM patients. Panel A shows a representative heatmap analysis of Western blotting quantification of indicated kinases phosphorylation upon treatment with 100 nM CXCL12 for 3 and 30 min. Data are expressed as normalized value of band intensity over untreated cells. Black square refers to undetectable signals. Heatmap plot was performed using pheatmap library.

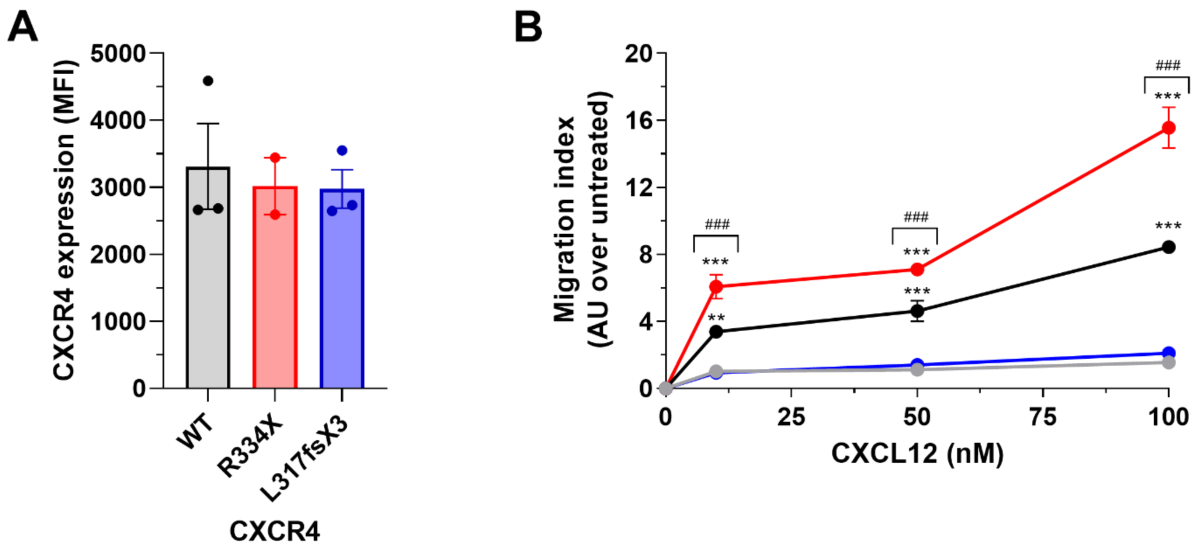

Figure S7. Functional properties of CXCR4 in K562 cell line expressing WT and WHIM-mutated variants.

Surface expression of CXCR4^{WT}, CXCR4^{R334X} and CXCR4^{L317fsX3} in K562 transient transfectants (A) tested for cell migration upon treatment with CXCL12 (B). Data are expressed as migration index over untreated and are shown as mean ± SEM of 2 independent experiments. CXCR4^{WT}: black; CXCR4^{R334X}: red; CXCR4^{L317fsX3}: blue; untreated: gray **: $p < 0.005$, ***: $p < 0.0001$: WHIM-mutated variants to WT, $^{\#\#\#}$: p < 0.0001: CXCL12 vs untreated.

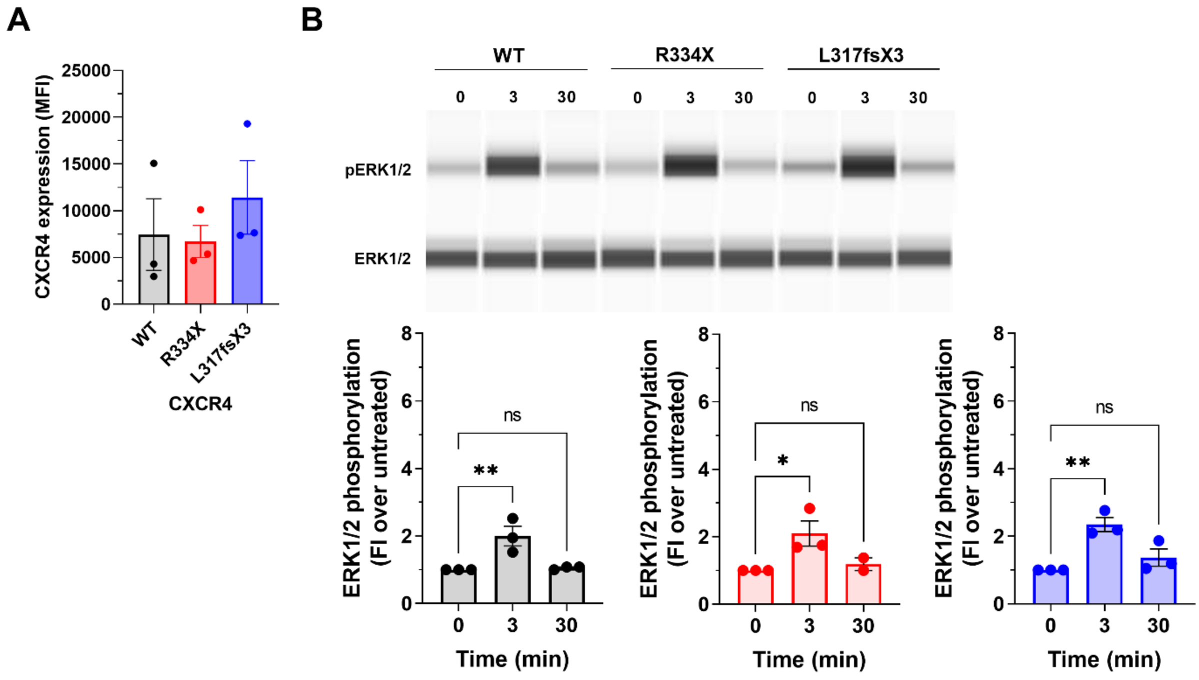

Figure S8. Signaling properties of CXCR4 in cell lines expressing WT and WHIM-mutated variants. Surface expression of $CXCR4^{WT}$, $CXCR4^{R334X}$ and $CXCR4^{L317fsX3}$ in HEK-293T transient transfectants (A) tested for pERK1/2 phosphorylation (B). Western blot analysis and quantification of ERK1/2 phosphorylation upon treatment with 100 nM CXCL12 for indicated time points. Data are shown as mean ± SEM of 3 independent experiments. *: $p < 0.05$, **: $p < 0.001$. Healthy donor: black bars; $CXCR4^{L317fsX3}$WHIM patient: blue bars.

PDF OF FOLLOWING FIGURES ARE UPLOADED AS SEPARATED FILES

Figure S4. CXCR4^{WT} signaling network, at T3 and T30, reconstructed from phosphoproteins measured in the phospho-proteins array. Node color code (from blue to red) correlates with fold change, while edge color code indicates Activation (Blue), Inhibition (red) and Docking (green).

Figure S5. CXCR4^{R334X} signaling network, at T3 and T30, reconstructed from phosphoproteins measured in the phospho-proteins array. Node color code (from blue to red) correlates with fold change, while edge color code indicates Activation (Blue), Inhibition (red) and Docking (green).

Figure S6. CXCR4^{L371fsX3} signaling network, at T3 and T30, reconstructed from phosphoproteins measured in the phospho-proteins array. Node color code (from blue to red) correlates with fold change, while edge color code indicates Activation (Blue), Inhibition (red) and Docking (green).

Figure S9. Functional and topological analysis of the CXCR4 signaling network reconstructed from phosphoproteins measured in the phospho-proteins array.

A) KEGG pathways enriched by considering the phosphoproteins measured in our work. KEGG pathway were retrieved by Functional Annotation Tool of DAVID database (background = Homo sapiens, count > 10, $p < 0.001$ and FDR<0.01). B) Violin plot reporting the average Betweenness in +CXCR4 and -CXCR4 random network models; the average Betweenness value in reference networks is shown, too. C) Interference values by comparing +CXCR4 and - CXCR4 networks (Interference= (+CXCR4_Betweenness) - (-CXCR4_Betweenness)).

SUPPLEMENTARY TABLE AND TABLE LEGENDS

PDF OF FOLLOWING TABLES ARE UPLOADED AS SEPARATED FILES

Table S1. Full immunophenotype of CXCR4^{L317fsX3} WHIM patients.

Table S2. List of phosphoproteins considered in the phospho-proteins array.

For each phosphoprotein the Original (commercial) name, the Official Gene Name and the fold change value in CXCR4^{WT} and the WHIM-mutated variants R334X and L317faX3, at T3 and T30, are shown.

Table S3. Topological analysis and centralities value in CXCR4 signaling network reconstructed from phosphoproteins measured in the phospho-proteins array.

Betweenness, Bridging, Centroid and Interference ((Interference= (+CXCR4_Betweenness) - (-CXCR4_Betweenness)) values are shown; specifically, centralities were calculated by considering the reconstructed signaling network with (+CXCR4) and without (-CXCR4) the presence of CXCR4.

PRIMERS' LIST

**PRIMER**	**SEQUENCE**
EX 1 For	5′- CAG CAG GTA GCA AAG TGA CG -3′
EX 1 Rev	5′- ACT CCT TTC GGT GAC CCT TT -3′
EX 2a For	5′- AAA GAG GAG GTC GGC CAC T -3′
EX 2a Rev	5′- GCT TGC TGA ATT GGA AGT GA -3′
EX 2b For	5′- ATG GAT TGG TCA TCC TGG TC -3′
EX 2b Rev	5′- CTC ACT GAC GTT GGC AAA GA -3′
EX 2c For	5′- GGT GGT CTA TGT TGG CGT CT -3′
EX 2c Rev	5′- CTC ACA CCC TTG CTT GAT -3′
EX 2d For	5′- GGA TCA GCA TCG ACT CCT TC -3′
EX 2d Rev	5′- AAA TCC AAC AAG CAA TAA AAA CTG -3′

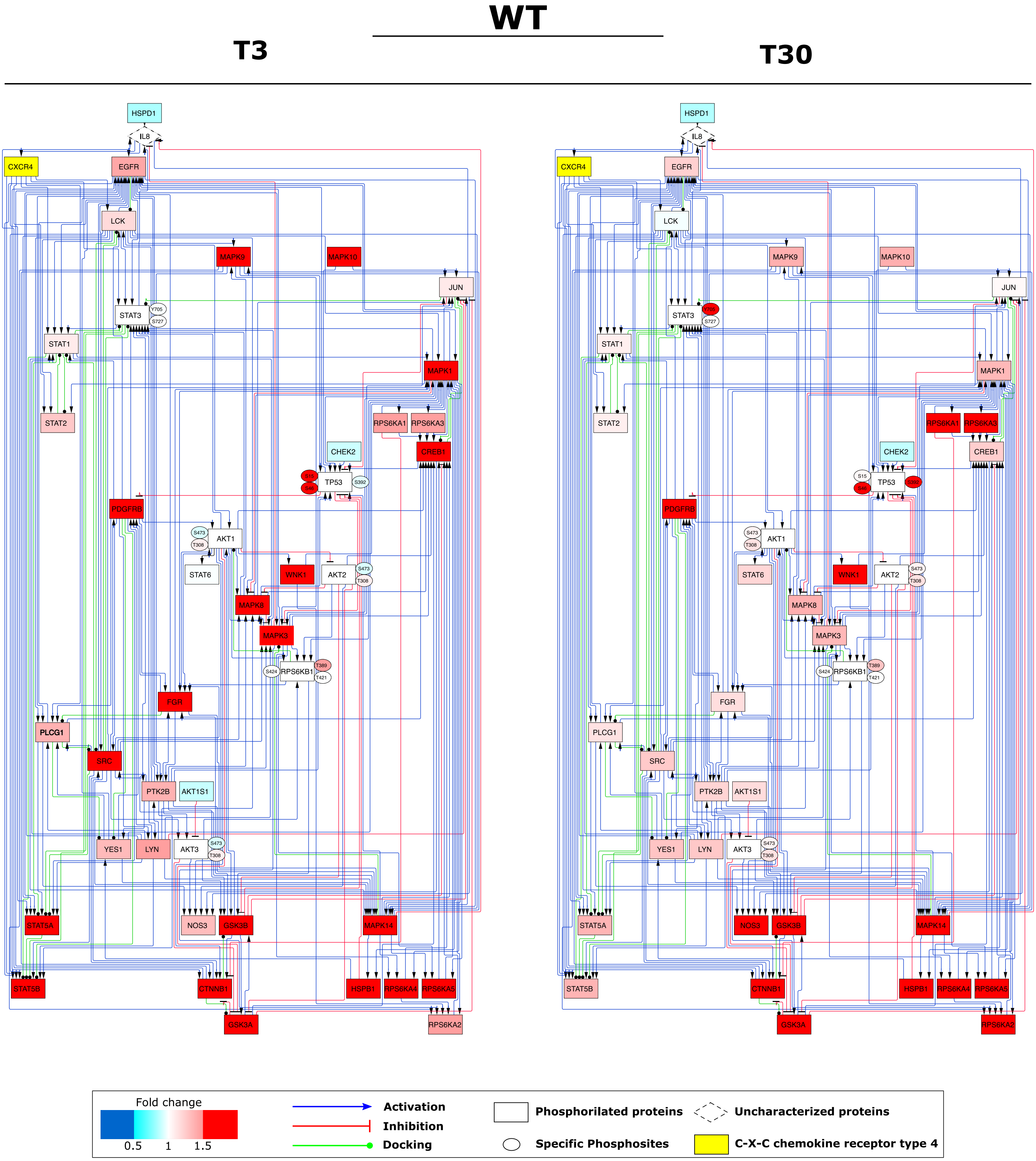

WT
T3
T30
HSPD1
IL8
CXCR4
EGFR
LCK
MAPK9
MAPK10
JUN
STAT3
Y705
S727
STAT1
MAPK1
STAT2
RPS6KA1
RPS6KA3
CHEK2
CREB1
TP53
S15
S46
S392
PDGFRB
AKT1
S473
T308
STAT6
WNK1
AKT2
MAPK8
MAPK3
RPS6KB1
S424
T389
T421
FGR
PLCG1
SRC
PTK2B
AKT1S1
YES1
LYN
AKT3
STAT5A
NOS3
GSK3B
MAPK14
STAT5B
CTNNB1
HSPB1
RPS6KA4
RPS6KA5
GSK3A
RPS6KA2
Fold change
0.5
1
1.5
Activation
Inhibition
Docking
Phosphorilated proteins
Specific Phosphosites
Uncharacterized proteins
C-X-C chemokine receptor type 4

R334X

T3 T30

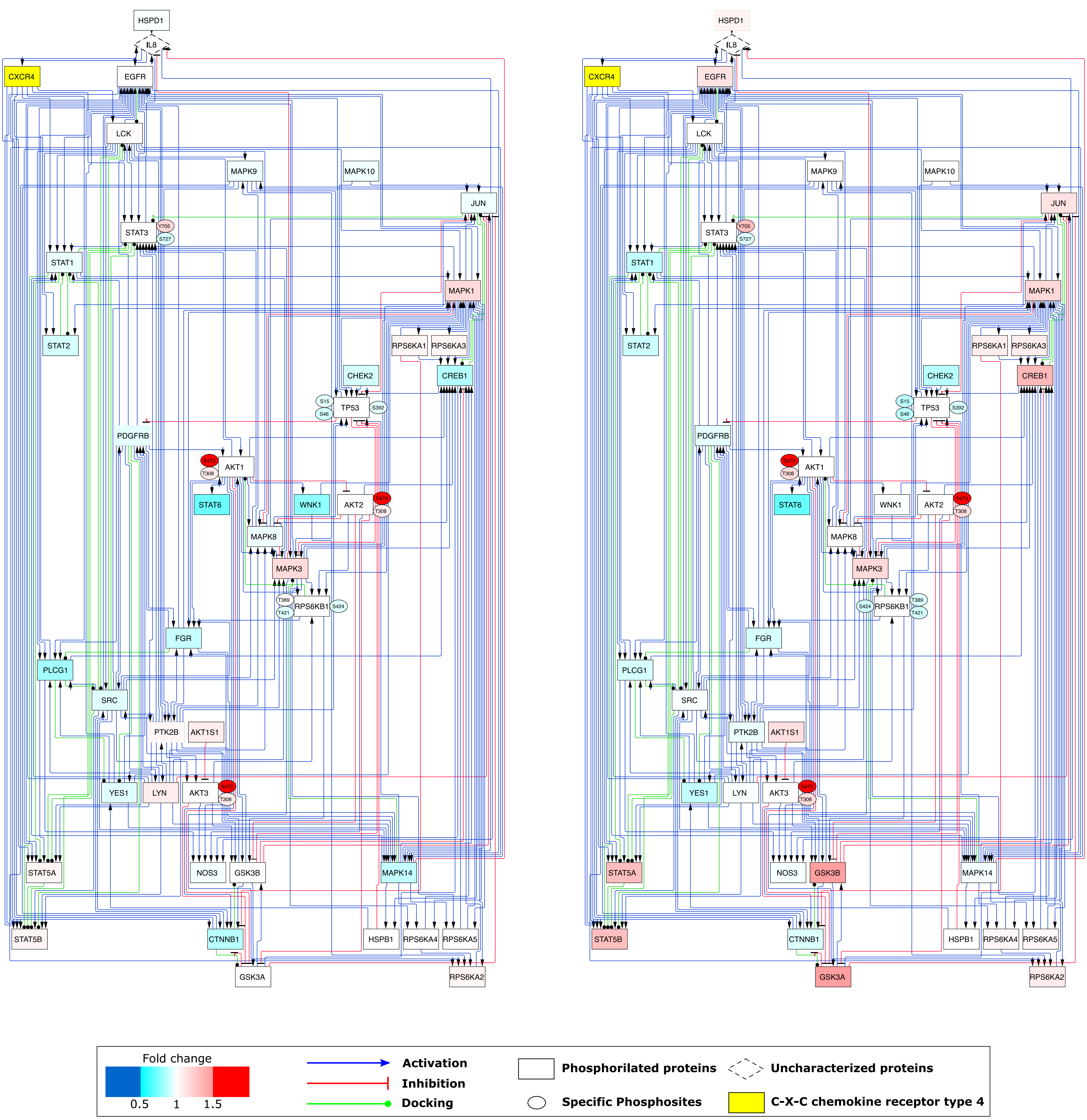

HSPD1
IL8
CXCR4
EGFR
LCK
MAPK9
MAPK10
JUN
STAT3
Y705
S727
STAT1
MAPK1
STAT2
RPS6KA1
RPS6KA3
CHEK2
CREB1
S15
S46
TP53
S392
PDGFRB
S473
T308
AKT1
STAT6
WNK1
AKT2
MAPK8
MAPK3
T389
T421
RPS6KB1
S424
FGR
PLCG1
SRC
PTK2B
AKT1S1
YES1
LYN
AKT3
STAT5A
NOS3
GSK3B
MAPK14
STAT5B
CTNNB1
HSPB1
RPS6KA4
RPS6KA5
GSK3A
RPS6KA2
Fold change
0.5
1
1.5
Activation
Inhibition
Docking
Phosphorilated proteins
Specific Phosphosites
Uncharacterized proteins
C-X-C chemokine receptor type 4

L317fsX3

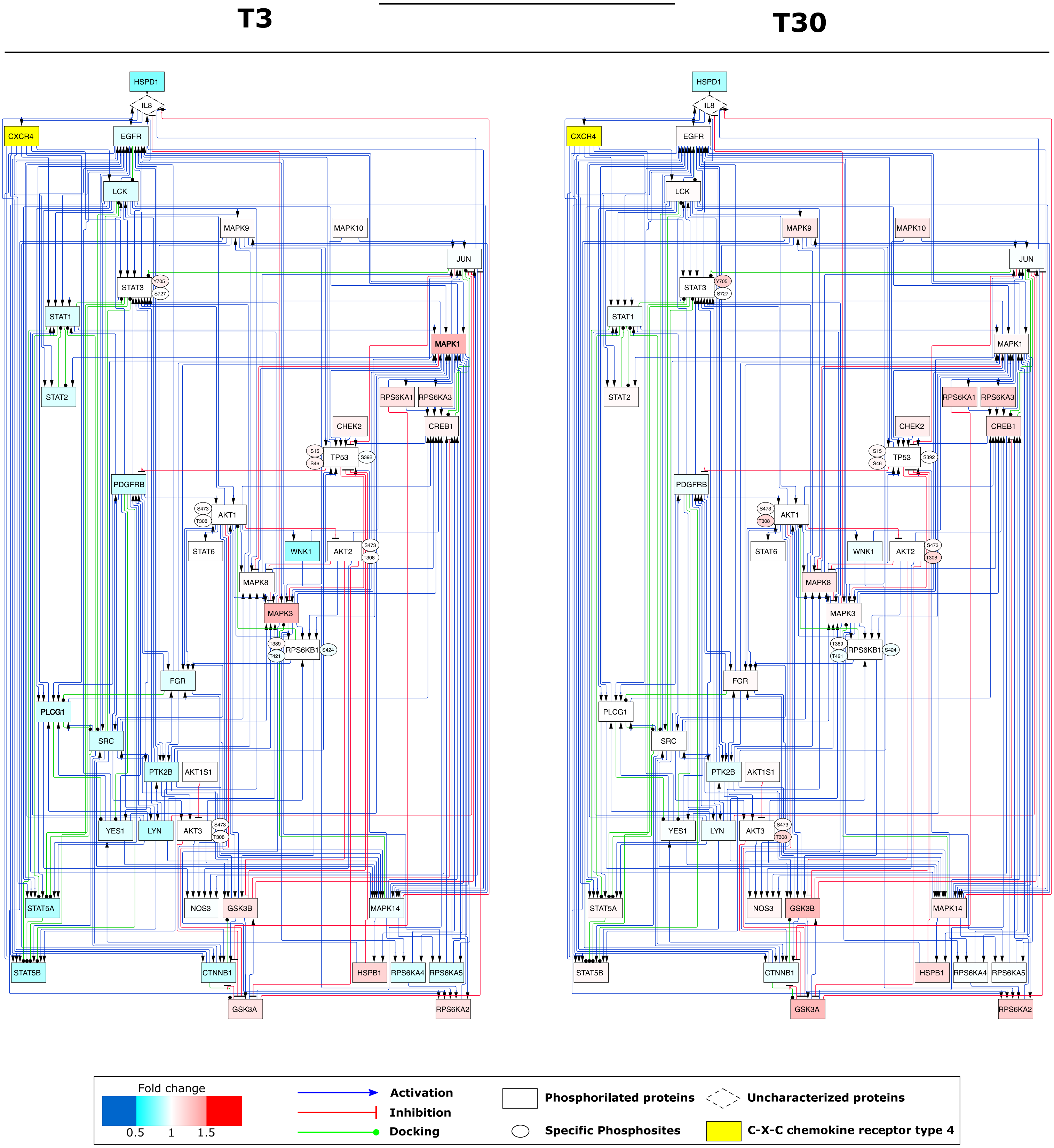
T3
T30
HSPD1
IL8
CXCR4
EGFR
LCK
MAPK9
MAPK10
JUN
STAT3
Y705
S727
STAT1
MAPK1
STAT2
RPS6KA1
RPS6KA3
CHEK2
CREB1
S15
S46
TP53
S392
PDGFRB
S473
T308
AKT1
STAT6
WNK1
AKT2
MAPK8
MAPK3
T389
T421
RPS6KB1
S424
FGR
PLCG1
SRC
PTK2B
AKT1S1
YES1
LYN
AKT3
STAT5A
NOS3
GSK3B
MAPK14
STAT5B
CTNNB1
HSPB1
RPS6KA4
RPS6KA5
GSK3A
RPS6KA2
Fold change
0.5
1
1.5
Activation
Inhibition
Docking
Phosphorilated proteins
Specific Phosphosites
Uncharacterized proteins
C-X-C chemokine receptor type 4

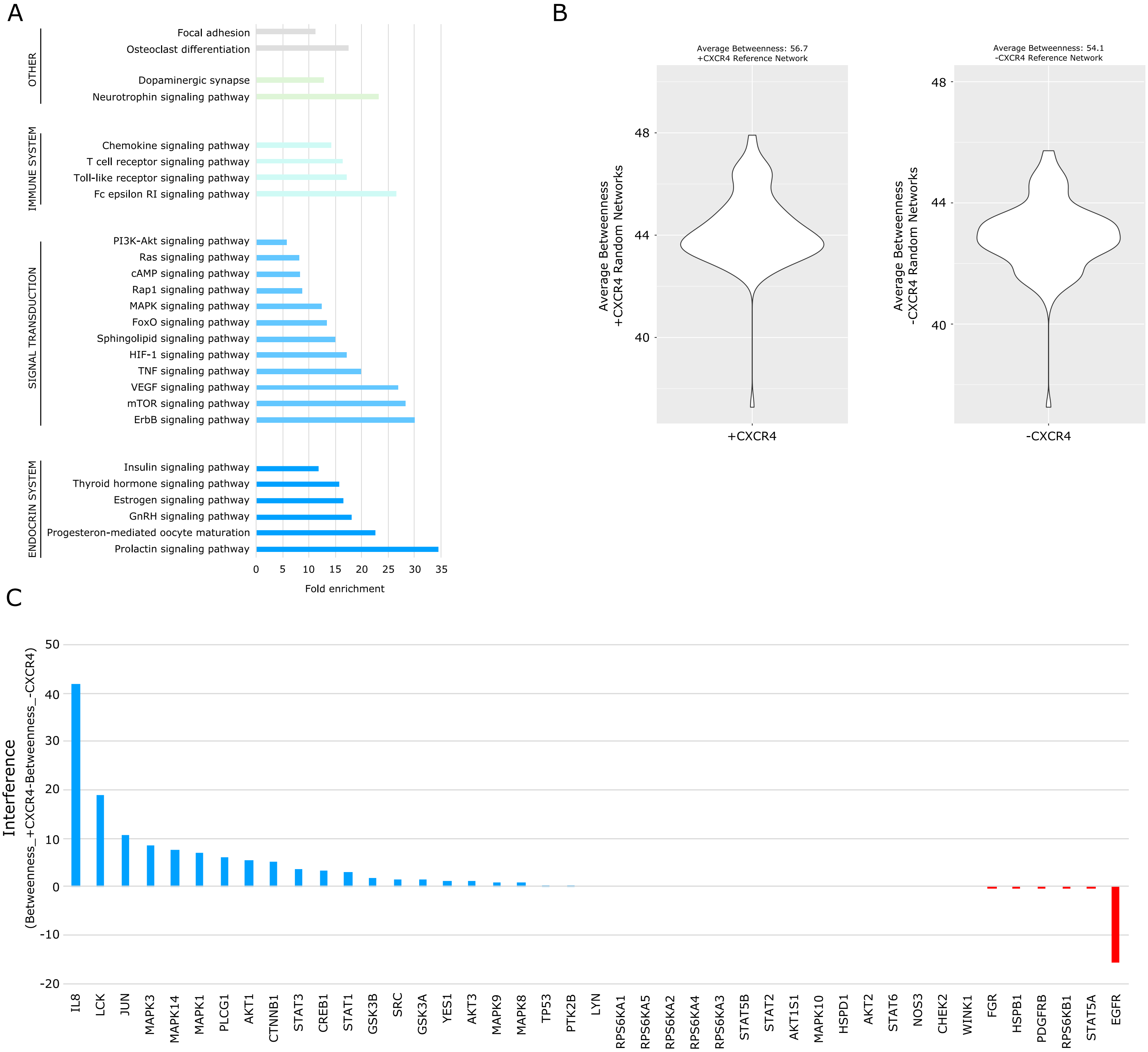
A
OTHER
Focal adhesion
Osteoclast differentiation
Dopaminergic synapse
Neurotrophin signaling pathway
IMMUNE SYSTEM
Chemokine signaling pathway
T cell receptor signaling pathway
Toll-like receptor signaling pathway
Fc epsilon RI signaling pathway
SIGNAL TRANSDUCTION
PI3K-Akt signaling pathway
Ras signaling pathway
cAMP signaling pathway
Rap1 signaling pathway
MAPK signaling pathway
FoxO signaling pathway
Sphingolipid signaling pathway
HIF-1 signaling pathway
TNF signaling pathway
VEGF signaling pathway
mTOR signaling pathway
ErbB signaling pathway
ENDOCRIN SYSTEM
Insulin signaling pathway
Thyroid hormone signaling pathway
Estrogen signaling pathway
GnRH signaling pathway
Progesteron-mediated oocyte maturation
Prolactin signaling pathway
0 5 10 15 20 25 30 35
Fold enrichment
B
Average Betweenness: 56.7
+CXCR4 Reference Network
Average Betweenness
+CXCR4 Random Networks
48
44
40
+CXCR4
Average Betweenness: 54.1
-CXCR4 Reference Network
Average Betweenness
-CXCR4 Random Networks
48
44
40
-CXCR4
C
Interference
(Betweenness_+CXCR4-Betweenness_-CXCR4)
50
40
30
20
10
0
-10
-20
IL8
LCK
JUN
MAPK3
MAPK14
MAPK1
PLCG1
AKT1
CTNNB1
STAT3
CREB1
STAT1
GSK3B
SRC
GSK3A
YES1
AKT3
MAPK9
MAPK8
TP53
PTK2B
LYN
RPS6KA1
RPS6KA5
RPS6KA2
RPS6KA4
RPS6KA3
STAT5B
STAT2
AKT1S1
MAPK10
HSPD1
AKT2
STAT6
NOS3
CHEK2
WINK1
FGR
HSPB1
PDGFRB
RPS6KB1
STAT5A
EGFR
