# Supplementary material for: Reduced G protein signaling despite impaired internalization and β-arrestin recruitment in patients carrying a CXCR4Leu317fsX3 mutation causing WHIM syndrome
Source: JCI Insight. 2023 Mar 8;8(5):e145688. doi: 10.1172/jci.insight.145688 (PMC10077478; doi:10.1172/jci.insight.145688)
Supplement: Supplemental table 1 [file jciinsight-8-145688-s243.pdf]

**Patient**	**P19**		**P20**		**P21**	
**Age at diagnosis** (years)	11		4		33	
**Total lymphocytes** (normal range)	1650 cells/µl (1000-5300)		1800 cells/µl (1700-6900)		1000 cells/µl (1000-2800)	
	%	*cells/µl*	*%*	*cells/µl*	*%*	*cells/µl*
**T cells** (CD3+)	68.0 (58.1-78.6)	1121 (1000-5300)	78.0 (60.5-79.8)	1404 (900-4500)	86.9 (66.6-86.1)	869 (700-2100)
**CD3+CD4+**	40.6 (27.3-48.5)	**669** (800-3500)	65.9 (30.3-48.3)	1186 (500-2400)	43.1 (31.7-60.2)	431 (300-1400)
CD4+ HLA-DR+	**0.6** (1.4-17.6)	4	**0.7** (1.4-17.6)	8	2.1 (1.4-17.6)	9
CD4+ naive (CD45RA+CCR7+)	**31.2** (53.6-81.4)	209	64.5 (34.3-74.6)	765	19.9 (17.2-61.0)	86
CD4+ RTE (CD45RA+CCR7+CD31+)	**26.3** (41.7-72.8)	176	56.2 (21.1-63.5)	667	17.4 (9.5-51.9)	75
CD4+ central memory (CD45RA-CCR7+)	21.6 (12.1-24.5)	145	16.0 (13.0-43.5)	190	25.6 (14.2-43.1)	110
CD4+ effector memory (CD45RA-CCR7-)	**42.8** (5.1-17.9)	285	15.1 (8.5-28.1)	179	52.3 (15.3-58.2)	225
CD4+ terminally differentiated (CD45RA+CCR7-)	4.2 (1.0-9.5)	28	4.3 (0.7-6.6)	51	2.3 (0.4-7.0)	10
**CD3+CD8+**	23.4 (15.0-32.7)	**386** (400-2100)	**8.2** (13.8-37.5)	**148** (300-1600)	**41.2** (14.7-39.4)	412 (200-900)
CD8+ HLA-DR+	**1.6** (2.1-52.0)	6	4.0 (2.1-52.0)	6	1.5 (2.1-52.0)	6
CD8+ naive (CD45RA+CCR7+)	**4.9** (23.0-85.1)	19	60.4 (26.7-72.9)	89	4.5 (4.1-63.0)	19
CD8+ central memory (CD45RA-CCR7+)	0.7 (0.2-7.6)	3	2.2 (1.2-11.6)	3	1.4 (0.2-18.4)	6
CD8+ effector memory (CD45RA-CCR7-)	**68.6** (7.0-37.8)	265	22.3 (6.0-53.6)	33	**64.8** (7.8-52.8)	267
CD8+ terminally differentiated (CD45RA+CCR7-)	25.2 (4.0-71.1)	97	15.2 (3.9-72.0)	22	29.4 (16.8-64.0)	121
CD4+CD8+	0.3 (0.2-3.1)	4	**0.3** (0.1-3.1)	6	0.7 (0.1-3.1)	7
CD4-CD8-	3.8 (1.9-25.8)	62	3.6 (1.9-25.8)	64	**1.8** (1.9-25.8)	18
TCR γ/δ	2.3 (0.5-21.5)	38	1.7 (0.5-21.5)	31	**0.3** (0.5-21.5)	3
**B cells** (CD19+)	**3.7** (9.8-28.0)	**61** (200-600)	**4.8** (5.7-19.7)	**87** (600-3100)	**2.5** (4.2-11.7)	**25** (100-500)
Recent bone marrow emigrants (CD38^{hi}CD21$^{dim/lo}$CD27-)	**59.6** (19.3-43.0)	36	**55.8** (15.0-35.3)	48	12.6 (1.8-23.8)	3
Naive (CD38$^{dim/lo}$CD21^{hi}CD27-)	**21.8** (40.6-57.9)	13	**28.8** (33.8-79.6)	25	33.0 (29.0-70.1)	8
CD19^{hi}CD21^{lo}	6.9 (2.3-9.0)	4	5.0 (1.1-10.0)	4	11.9 (1.4-19.9)	3
Switched memory (IgD-CD27+)	4.0 (1.8-10.5)	2	**0.9** (2.7-20.6)	1	26.3 (7.2-26.3)	7
IgM memory (IgD+CD27+)	5.4 (4.9-13.4)	3	4.9 (3.5-24.1)	4	15.2 (8.2-30.0)	4
Terminally differentited (CD38^{hi}CD27^{hi}CD21^{lo})	**0.27** (0.99-7.40)	0	1.09 (0.16-8.70)	1	1.07 (0.09-5.82)	0
**NK cells** (CD3-CD16+CD56+)	26.7 (5.0-28.4)	440 (70-1200)	15.1 (4.6-27.8)	271 (100-1400)	9.9 (4.4-23.6)	99 (90-600)
