# Supplementary material for: Reduced G protein signaling despite impaired internalization and β-arrestin recruitment in patients carrying a CXCR4Leu317fsX3 mutation causing WHIM syndrome
Source: JCI Insight. 2023 Mar 8;8(5):e145688. doi: 10.1172/jci.insight.145688 (PMC10077478; doi:10.1172/jci.insight.145688)
Supplement: Supplemental table 2 [file jciinsight-8-145688-s244.pdf]

ANNOTATIONS			FOLD ENRICHMENT					
Original Name	Official Gene Name	Specific Phosphosite	WT T3	WT T30	R334X T3	R334X T30	L317AAX T3	L317AAX T30
AKT1	AKT1	S473	0,93	1,06	1,91	3,08	1,01	1,03
AKT1	AKT1	T308	1,08	1,14	1,10	1,17	1,01	1,19
AKT2	AKT2	S473	0,93	1,06	1,91	3,08	1,01	1,03
AKT2	AKT2	T308	1,08	1,14	1,10	1,17	1,01	1,19
AKT3	AKT3	S473	0,93	1,06	1,91	3,08	1,01	1,03
AKT3	AKT3	T308	1,08	1,14	1,10	1,17	1,01	1,19
c-Jun	JUN		1,11	1,03	0,97	1,14	0,99	0,99
Chk-2	CHEK2		0,90	0,88	0,92	0,86	1,07	1,09
CREB	CREB1		1,73	1,24	0,85	1,35	1,07	1,17
EGFR	EGFR		1,43	1,23	1,01	1,14	0,94	1,02
eNOS	NOS3		1,33	1,72	0,98	0,98	0,99	1,05
ERK1	MAPK3		2,00	1,35	1,18	1,19	1,37	1,04
ERK2	MAPK1		2,00	1,35	1,18	1,19	1,37	1,04
fgr	FGR		1,55	1,16	0,89	0,92	0,94	1,03
GSK-3α\β	GSK3A		1,58	3,87	1,00	1,47	1,13	1,34
GSK-3β	GSK3B		1,58	3,87	1,00	1,47	1,13	1,34
HSP27	HSPB1		2,94	2,16			1,21	1,18
HSP60	HSPD1		0,84	0,85	0,99	1,06	0,75	0,84
JNK1	MAPK8		1,53	1,39	0,97	1,01	1,02	1,12
JNK2	MAPK9		1,53	1,39	0,97	1,01	1,02	1,12
JNK3	MAPK10		1,53	1,39	0,97	1,01	1,02	1,12
Lck	LCK		1,18	0,98	1,02	1,01	0,93	1,02
Lyn	LYN		1,47	1,27	1,09	0,99	0,87	0,97
MSK1	RPS6KA5		1,92	1,80	1,01	1,04	0,92	0,99
MSK2	RPS6KA4		1,92	1,80	1,01	1,04	0,92	0,99
P38α	MAPK14		2,06	1,69	0,88	0,98	0,97	1,10
P53 (S15)	P53	S15	2,03	1,03	0,95	0,87	1,06	1,03
P53 (S392)	P53	S392	0,95	1,99	0,95	0,93	0,99	1,01
P53 (S46)	P53	S46	1,71	1,53	0,92	0,87	1,07	1,05
p70 S6 Kinase (T389)	RPS6KB1	T389	1,47	1,29	1,02	0,93	1,04	1,02
p70 S6 Kinase (T421\S424)	RPS6KB1	T421			0,94	0,91	0,97	0,97
p70 S6 Kinase (T421\S424)	RPS6KB1	S424			0,94	0,91	0,97	0,97
PDGF Rβ	PDGFRB		1,78	1,51	0,95	0,95	0,90	0,97
PLC-γ1	PLCG1		1,38	1,14	0,82	0,92	0,91	1,00
PRAS40	AKT1S1		0,89	1,17	1,09	1,15	1,01	1,02
PYK2	PTK2B		1,38	1,21	1,04	0,96	0,89	0,95
RSK1	RPS6KA1		1,47	1,80	1,05	1,09	1,14	1,24
RSK2	RPS6KA3		1,47	1,80	1,05	1,09	1,14	1,24
RSK3	RPS6KA2		1,47	1,80	1,05	1,09	1,14	1,24
Src	SRC		1,70	1,26	0,94	0,99	0,91	1,00
STAT1	STAT1		1,10	1,08	0,96	0,88	0,93	0,98
STAT2	STAT2		1,27	1,08	0,92	0,90	0,93	1,03
STAT3	STAT3	Y705	0,99	1,67	1,20	1,32	1,09	1,23
STAT3	STAT3	S727			0,93	0,94		
STAT5b	STAT5A		1,51	1,37	1,05	1,32	0,85	1,04
STAT5a	STAT5B		1,51	1,37	1,05	1,32	0,85	1,04
STAT6	STAT6		0,99	1,20	0,71	0,70		
WNK1	WNK1		1,69	1,87	0,78	0,99	0,80	0,97
Yes	YES1		1,34	1,29	0,94	0,88	0,94	0,99
β-Catenin	CTNNB1		1,69	1,56	0,86	0,93	0,89	0,96
