# Supplementary material for: Reduced G protein signaling despite impaired internalization and β-arrestin recruitment in patients carrying a CXCR4Leu317fsX3 mutation causing WHIM syndrome
Source: JCI Insight. 2023 Mar 8;8(5):e145688. doi: 10.1172/jci.insight.145688 (PMC10077478; doi:10.1172/jci.insight.145688)
Supplement: Supplemental table 3 [file jciinsight-8-145688-s245.pdf]

	Betweenness			Bridging		Centroid	
Gene Name	+CXCR4	-CXCR4	Interference	+CXCR4	-CXCR4	+CXCR4	-CXCR4
EGFR	280,1	295,7	-15,6	8,2	8,3	-37	-36
MAPK1	270,6	263,7	6,8	2,7	2,6	-37	-36
TP53	255,7	255,5	0,2	8,8	8,8	-37	-36
PDGFRB	150,4	150,7	-0,3	8,1	7,8	-37	-36
MAPK3	147,3	138,9	8,4	3,7	3,4	-37	-36
CREB1	125,2	121,9	3,3	3,4	3,3	-37	-36
AKT1	118,9	113,5	5,4	3,5	3,3	-37	-36
SRC	114,5	113,1	1,4	3,5	3,3	-37	-36
MAPK14	104,5	96,8	7,7	2,7	2,4	-37	-36
IL8	100,2	58,2	42,0	8,4	6,0	-37	-36
FGR	85,3	85,3	0,0	12,5	12,4	-37	-36
STAT1	84,7	81,9	2,8	4,0	3,9	-37	-36
CTNNB1	83,5	78,4	5,0	7,6	7,0	-37	-36
RPS6KB1	71,0	71,4	-0,4	12,5	12,6	-37	-36
GSK3B	60,9	59,3	1,6	3,6	3,5	-37	-36
JUN	55,4	44,8	10,6	2,2	1,8	-37	-36
LCK	53,8	35,0	18,8	2,2	1,5	-37	-36
PTK2B	49,4	49,3	0,0	3,1	3,1	-37	-36
STAT3	44,8	41,3	3,6	3,0	3,2	-37	-36
AKT3	40,2	39,2	1,0	4,4	4,3	-37	-36
MAPK8	36,4	35,7	0,7	2,7	2,6	-37	-36
PLCG1	31,3	25,2	6,1	2,5	2,5	-37	-36
YES1	30,7	29,6	1,1	4,1	3,9	-37	-36
GSK3A	15,1	13,8	1,3	2,0	1,8	-37	-36
MAPK9	13,2	12,2	1,0	2,6	2,4	-37	-36
LYN	5,4	5,4	0,0	0,5	0,4	-37	-36
RPS6KA1	4,3	4,3	0,0	4,6	4,6	-37	-36
HSPB1	2,0	2,0	0,0	2,7	2,7	-37	-36
RPS6KA5	1,5	1,5	0,0	1,2	1,2	-37	-36
STAT5A	0,7	1,1	-0,4	0,1	0,2	-1	-1
RPS6KA2	0,6	0,6	0,0	0,2	0,2	-37	-36
RPS6KA4	0,5	0,5	0,0	0,2	0,2	-37	-36
RPS6KA3	0,2	0,2	0,0	1,1	1,1	-37	-36
STAT5B	0,0	0,0	0,0	0,0	0,0	0	0
STAT2	0,0	0,0	0,0	0,0	0,0	-37	-36
AKT1S1	0,0	0,0	0,0	0,0	0,0	-38	-37
MAPK10	0,0	0,0	0,0	0,0	0,0	-38	-37
HSPD1	0,0	0,0	0,0	0,0	0,0	-38	-37
AKT2	0,0	0,0	0,0	0,0	0,0	-37	-36
STAT6	0,0	0,0	0,0	0,0	0,0	0	0
NOS3	0,0	0,0	0,0	0,0	0,0	0	0
CHEK2	0,0	0,0	0,0	0,0	0,0	-38	-37
WNK1	0,0	0,0	0,0	0,0	0,0	-38	-37
